# Effect of the health and wellness Kneipp concept on health promotion and reduction of sick days for kindergarten children: a cluster randomized controlled trial protocol

**DOI:** 10.3389/fmed.2024.1412971

**Published:** 2024-07-26

**Authors:** Marinela Gerganova, Steven Schepanski, Martin Bogdanski, Farid I. Kandil, Angela Tekath, Michael Jeitler, Wiebke Stritter, Sarah B. Blakeslee, Georg Seifert

**Affiliations:** ^1^Charité Competence Center for Traditional and Integrative Medicine (CCCTIM), Charité – Universitätsmedizin Berlin, corporate member of Freie Universität Berlin and Humboldt – Universität zu Berlin, Berlin, Germany; ^2^Department of Pediatrics, Division of Oncology and Hematology, Charité – Universitätsmedizin Berlin, corporate member of Freie Universität Berlin and Humboldt – Universität zu Berlin, Berlin, Germany; ^3^Institute of Social Medicine, Epidemiology and Health Economics, Charité – Universitätsmedizin Berlin, corporate member of Freie Universität Berlin and Humboldt – Universität zu Berlin, Berlin, Germany; ^4^Department of Internal Medicine and Nature-Based Therapies, Immanuel Hospital Berlin, Berlin, Germany

**Keywords:** Kneipp, child health promotion, salutogenesis, kindergarten, hydrotherapy, lifestyle wellness, integrative medicine, common cold questionnaire

## Abstract

**Background:**

The holistic health and wellness Kneipp concept, has a long tradition in Europe with demonstrated health benefits. Based on the five elements of the Kneipp concept, kindergartens in and around Germany are used to certify “Kneipp Kindergartens” that practice regular Kneipp applications and activities: cold water applications, exercise, nutrition, herbs and mind-body interventions. Little is known about the potential health benefits for children, however. This study protocol describes our study design and intervention of the Kita Kneipp Study to investigate the effect of the Kneipp concept on kindergarten children aged 2–6 years.

**Methods and design:**

The Kita Kneipp Study, registered with the German Clinical Trial Register (DRKS-ID: DRKS00029275), is a confirmatory, mixed-method, two-armed, waitlist, clinical, cluster randomized controlled trial (RCT). Kindergartens in Berlin, Germany that would like to implement the Kneipp concept into their facility will be recruited and randomized to the intervention or control group. Changes in the number of kindergarten sick days will be the primary outcome measure. Kindergarten attendance and reason for absence including illness will be collected on a weekly basis at two time points for 6 weeks from the parents and kindergarten directors: baseline and 1 year after baseline. Secondary outcomes will measure cold symptoms through the Common Cold Questionnaire (CCQ) and National Cancer Institute – Common Terminology Criteria for Adverse Events (NCI-CTCAE) Scales describing gastroenterological-based symptoms Kindergarten educator sick days will be aggregately reported for the same time period. Kneipp concept activities will be recorded on a weekly basis over the one-year intervention period. To understand the experience of Kneipp concept implementation and possible changes in the kindergarten, expert interviews will be conducted with intervention kindergarten educators and focused ethnographies will be planned to observe and analyze the intervention activities.

**Discussion:**

This mixed method study design has potential to help identify if the Kneipp concept can be used for salutogenic purposes among young children and provide insights and experience of the implementation and practicing a holistic health and wellness concept in a kindergarten setting.

## 1 Introduction

**Figure 1:**
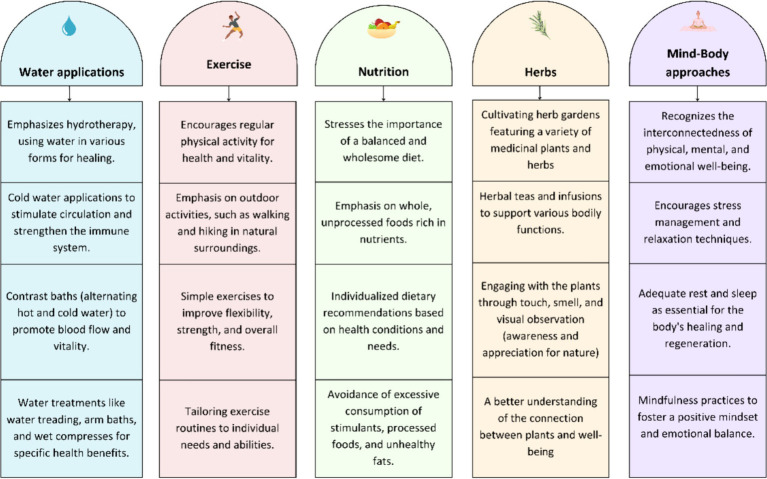
Five Elements of Kneipp.

Kneipp therapy is a holistic health, lifestyle and wellness approach that is embedded within German traditional medicine, based on complementary and naturopathy health concepts. The concept is named after the German priest and healer Sebastian Kneipp (1821–1897) who promoted the use of cold-water application from hydrotherapy in combination with a holistic health concept. Five principal elements are used for maintaining and sustaining health: temperature stimuli through cold (and warm) water applications, exercise, wholesome, plant-based, regional and seasonal nutrition, herbs and mind- body approaches (see Figure 1: Five Elements of Kneipp). This system, known as Kneipp therapy, is most well-known in German speaking countries and has one of the largest lay organizations for health and wellness in Germany (1). Kneipp therapy is used by trained and untrained practitioners in health promotion and for the prevention against diseases across a lifespan (2).

Childhood infection, especially in young children, creates a societal burden because a sick child at home requires a caregiver, who often must take a free day off work (3). Children and educators often experience cross-infections in kindergartens, contributing to the total burden of illness (4).

There is extensive research about the positive effects of a healthy diet and exercise on children’s overall health (5, 6). However, there is further research needed on Kneipp interventions especially with focus on the (cold) water applications and their effects on children’s health.

In a recent systematic review, evidence of the effects of Kneipp therapy across the life cycle were summarized; beneficial effects were noted for a wide range of conditions including varicose veins, menopausal syndrome, fever in children, absenteeism related to upper respiratory tract infections, hypertension, mild heart failure, sleep disturbances, cognition, and emotional functioning (7). The overall health benefits for children are inconclusive however, and few studies have been conducted with children and Kneipp therapy. Two pilot studies conducted with children aged 3–6 years that provide the foundation for this study found no significant reduction in infections through secretory IgA or fever but did demonstrate a lowered number of sick days for this sample (8, 9).

German kindergarten facilities, daycares through preschool system caring for young children between the ages of one to 6 years old, are currently being certified with a Kneipp concept that implements all five elements in the day care setting (10). Therefore, investigating whether a possible correlation exists between an implemented Kneipp concept and the number of absent sick days of children in such facilities would be of interest. For this purpose, we have planned a study with the aim to evaluate effects by monitoring absent sick days of children in kindergartens intending to implement a Kneipp concept and comparing this to data from kindergartens who are waitlisted to implement the Kneipp concept until after the study data has been collected. In order to evaluate the process of the concept-implementation and experiential dynamics, a qualitative approach will be used.

The following study questions will be examined:

– Quantitative hypothesis: Will the implementation of a child-friendly Kneipp concept in kindergartens improve health of kindergarten children aged 2–6 years, resulting in fewer illness-related absences from the kindergarten?
– Qualitative research question: How does the kindergarten-appropriate implementation of the holistic health and wellness Kneipp concept change the overall quality of work, dynamics, and atmosphere within the kindergarten?

## 2 Methods

The Kita Kneipp Study is a confirmatory, mixed-method, two-armed, waitlist, clinical, cluster RCT that was registered with the German Clinical Trial Register (DRKS-ID: DRKS00029275) on November 10, 2022 (submission for registration June 22, 2022), and approved by the Ethics Committee of the Charité - Universitätsmedizin Berlin (Application number: EA2/122/22, approved on September 13, 2022). It follows the standards of the Helsinki convention of good clinical practices.

This study protocol follows the SPIRIT (Standard Protocol Items: Recommendations for Interventional Trials) Guidelines for reporting study protocols (11).

### 2.1 Study setting

Prior to study data collection, the study team interviewed already-certified kindergarten directors to find out aspects of the Kneipp concept that worked well in the kindergarten setting or were potentially problematic. This expert input was used to adapt our study design to the recruited facilities.

Kindergartens that had interest in becoming certified as Kneipp kindergartens and whose personnel wanted to take part in an applied Kneipp training for kindergartens were to be recruited to the study through contacts at the Berlin Senate level who have access to a network of Berlin kindergartens. A requirement for participation is the willingness of the facilities to be randomized. Further, inclusion criteria stipulated that the kindergarten facilities be compatible for Kneipp interventions, for example that they had suitable shower facilities, outdoor spaces, and room for indoor exercise (12). Included kindergartens will be recruited in Berlin, Germany. Informational evenings are to be held with kindergarten directors, caregivers and personnel to inform about the project, and to answer questions about the study purposes, design, data protection and inclusion. Written consent for participation in the study is required from all caregivers and educators.

The study will be conducted with two-time measurement periods, each 6 weeks long. Parents will complete an online at the end of each week of the data collection period. At the beginning of each measurement period, parents will be asked to complete an initial onboarding questionnaire containing socioeconomic background information about the family. The first measurement period will be planned in autumn 2022, followed by a second measurement in the autumn of 2023. Kindergarten directors will also be asked to record absences and reasons for absence of the participating children and educators and report them after completion of each 6-week data collection period.

The quantitative data of the study will evaluate the number of sick days and symptoms for children aged 2–6 years in kindergartens that implement kindergarten-appropriate, Kneipp applications, compared to the kindergartens in the waitlist group.

### 2.2 Eligibility criteria

Eligibility crite shown in Table 1.

**Table 1:** Eligibility criteria.

	Inclusion criteria	Exclusion criteria
Kindergartens	Berlin kindergartens seeking certification as Kneipp daycare facilities with Kneipp-Bund e.V. Interested in participating in associated training via Sebastian Kneipp Academy. Willingness to engage in pair-randomized group assignment. Must provide pedagogical staff for training as Kneipp educators in autumn 2022 or 2023/24. Implementation of Kneipp concept immediately after training.	Kindergartens already implementing holistic health concepts (e.g., Kneipp, anthroposophical). Staff restricted from participating in training. Facilities unsuitable for implementing the Kneipp concept. Daycare center involved in another study preventing participation in this one.
Educators	Healthy educators, regardless of gender Working in the participating facilities 18–65 years of age Informed consent available	Serious chronic or acute illnesses inhibiting participation. Not speaking or understanding German
Caregivers	Caregivers, regardless of gender 18 years and older At least one child in a participating daycare center included in the study. Informed consent available	Serious chronic or acute illness preventing participation in the data collection. Not speaking or understanding German at the appropriate level for reading and completing study information
Children	Healthy children, regardless of gender 2–6 years Care in the participating kindergartens during study period Consent of the caregivers	Serious chronic or acute illness Not speaking or understanding German Age under 2y/o

### 2.3 Interventions and blinding

Prior to being randomized into the group, kindergartens will be matched-paired according to socio-demographic information for Berlin neighborhoods and address (13, 14). Then kindergarten pairs will be cluster-randomized via computer randomization program by a team member without prior or background knowledge of kindergartens into the immediate start or waitlist group. Once they are allocated by the randomizing computer program into intervention or waitlist groups, they will be informed which group they are in. If a kindergarten declines to take part in the study based on group allocation, another replacement kindergarten will be selected for the recruitment vacancy.

#### 2.3.1 Intervention group

**Figure 2:**
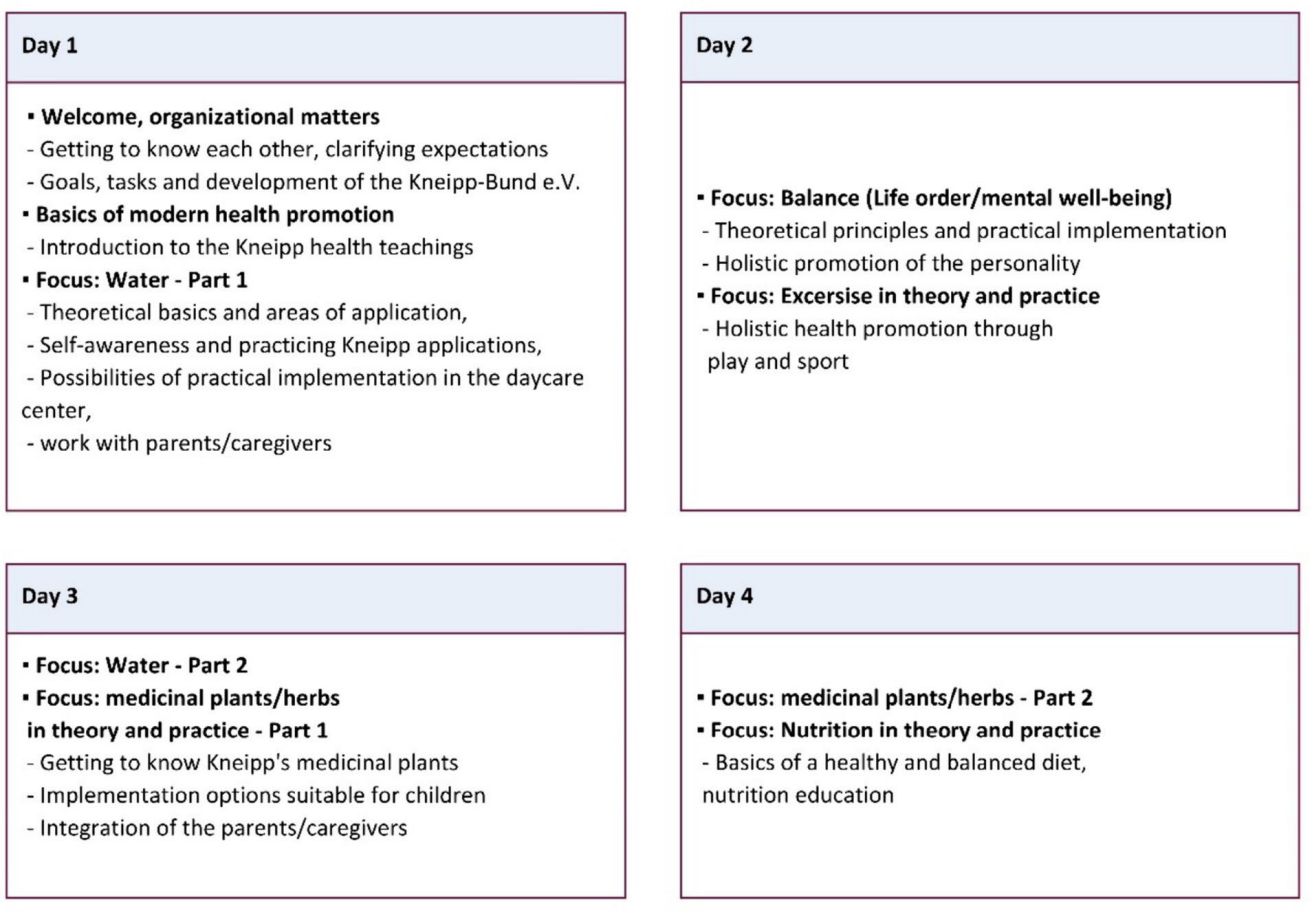
Curriculum overview. Kneipp Educator Training.

First, the kindergarten educators of the intervention group will receive a planned 4-day (40 h) training of the Kneipp concept (see Figure 2: Curriculum Overview. Kneipp Educator Training) (10). The training will be focused on integrating the Kneipp concept with young children in the kindergarten setting with the 5 elements. Educators from intervention facilities will be trained together to allow an exchange among participants, enhance collective learning, share insights, and foster an environment for discussions. The training program will be conducted by two experienced trainers from Kneipp-Bund e.V.: one with expertise in hydrotherapy and mind–body practices, and the other specialized in herbs and nutrition.

The waitlist group will also complete the training after the last data collection period. While there will be project mandate as to which interventions must be conducted, the educators in the intervention group will be encouraged to commence interventions directly after the training, particularly with emphasis and daily practice of cold-water applications. (see Figure 1: Five Elements of Kneipp) Intervention activities will be reported on a weekly basis by the kindergartens and recorded by the team. Furthermore, a qualitative methodology is employed to evaluate whether introducing the Kneipp concept will affect factors such as stress levels, quality of work in the kindergarten environment, interactions among children and educators. This involves conducting focused ethnography (15) on two separate days (one in spring/summer and one in autumn/winter) to obtain a better overview of the applications used in different seasons. Semi-structured interviews will be conducted with educators and kindergarten directors to understand and evaluate the use of the interventions, as well as to detect possible changes among kindergarten group dynamics and kindergarten educators. Maximum variation sampling strategy will be applied to provide rich and diverse perspectives, used for answering the qualitative research question. All kindergarten directors and at least 2 other educators from all the intervention facilities will be interviewed. The interviews will be conducted in the kindergarten facilities by a medical doctorate student (MG) until data saturation is reached. Throughout the process regular meetings with a group of qualitative researchers will be organized, where the means of data collection and analyzing process will be discussed, evaluated and reflected.

Another focus of the qualitative evaluation is to assess the feasibility of conducting larger multi-centric randomized studies in this context and the potential future implementation of hydrotherapy in kindergartens.

#### 2.3.2 Waitlist group

The educators in the waitlist group serving as the control will not begin practicing the Kneipp concept until after the second data collection period, after which they will participate in a training.

### 2.4 Outcomes

The primary outcome measure is the number of absent days due to illness among 2-6-year-old children in randomized Berlin kindergartens for the Kneipp concept intervention kindergartens compared to the matched waitlist kindergartens over two time points (baseline prior to the intervention and 12 months after baseline).

Additionally, the CCQ (16) and the NCI-CTCAE Scales (17) describing gastroenterological-based symptoms will be used as a secondary outcome measure at each of the two timepoints as well. The study will also gather data on the number of days educators are absent due to sickness. This information will be subjected to statistical analysis as an additional secondary outcome measure, aiming to identify potential correlations between the absence of educators and that of the children.

Summarized findings will identify categories and themes from the qualitative content analysis of observations and interviews highlighting the practice and experiences of implementing Kneipp in the kindergarten and perceived changes.

### 2.5 Sample size

The sample size selection was formed by a preceding non-randomized controlled study (8) which focused on kindergarten sick days as a primary outcome. The study employed a Chi2-Test (categorizing kindergarten sick days as yes/no), revealing an effect size of *ω* (omega) = 0.41, equivalent to a Cohen’s *d* = 0.66 (8). Recognizing the non-randomized nature of the prior trial, the effect size was conservatively adjusted in half to φ (phi) = 0.205. With standard parameters set at *α* (alpha) = 0.05 (indicating a 5% probability of error in two-sided testing for differences), a power of 80% (*β* (beta) = 0.20), and accounting for an estimated dropout rate of approximately 20%, the optimal sample size for the current study was determined to be 240 child participants. The number of recruited matched kindergarten pairs will be based on the total calculated sample size.

Using a sampling strategy of maximum variation, semi-structured qualitative expert interviews will be planned with up to 15 kindergarten educators in the intervention facilities, while two focused ethnography observations will be each of the intervention kindergartens.

### 2.6 Recruitment, randomization, and assignment of intervention

Gate keepers at the Berlin governing structures responsible for kindergartens, contact leads from cooperative partners in Berlin will identify interested kindergartens. Members of the study team (SB – experienced scientific researcher, study coordination, AT – study administration) will contact these via email in which a flyer about the project will be included as additional information. Once interested, kindergartens will be contacted via telephone and site feasibility will be assessed for inclusion.

After being recruited to the study, kindergartens with a similar socio-economic level will be match-paired according to statistical demographic analysis of locations in Berlin (14).

The kindergartens will then be randomized within their matched pair to either the intervention or the waitlist group using the randomizing function of Python 3.10.6, executed by an experienced biostatistician (FK). The allocated study group will not be blinded, as this would not be possible due to the nature of the interventions. Therefore, the kindergartens, caregivers and children will be aware whether they are in the Kneipp or in the waitlist group.

### 2.7 Data collection and management

The study will be conducted with two-time measurement periods, each 6 weeks long, whereby caregivers will complete an online questionnaire via online survey based on the CCQ and the NCI-CTCAE Scales describing gastroenterological-based symptoms at the end of each week of the data collection period.

**Figure 3:**
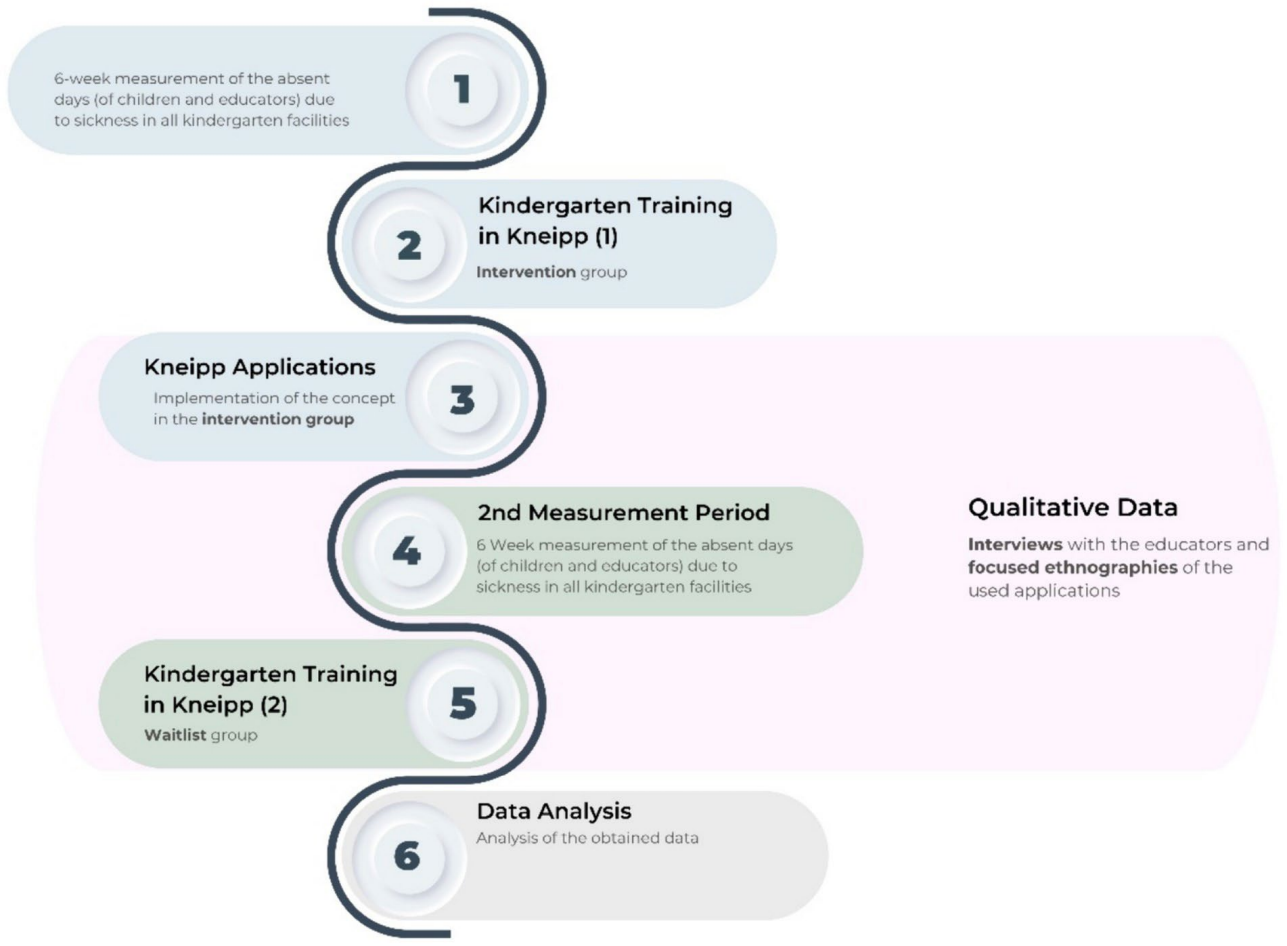
Data collection flowchart.

At the beginning of each measurement period, caregivers will be asked to complete an initial onboarding questionnaire containing socioeconomic background information about the family. The first measurement period will be planned in autumn 2022, followed by a second measurement in the autumn of 2023 (see Figure 3: Data Collection Flowchart).

All teachers and caregivers will be informed about the purpose and implementation of the data collection as part of the evaluation via printed information sheets. The educator’s and parent’s written consent to participate will be collected (on paper) by the kindergarten directors. The following data will be recorded: Name, gender, date of birth, email, telephone number. Consents will be filed in a project folder and stored in a lockable office belonging to the kindergarten directors. The folders will be periodically checked during site visits by the study team (SB, MG – medical doctoral student, study coordination).

Focused ethnographies will be planned with the kindergarten staff to ensure that visits coincide with planned interventions. Based on these visits, willing staff will be asked to participate in a separately scheduled expert interview. Interviews will be audio-recorded, auto-transcribed via f4x (audiotranskription.de), corrected and pseudonymized. Transcripts and observation protocols will be uploaded and analyzed on a single computer with the qualitative analysis software MAXQDA 24 (Release 24.2.0).

At the beginning of the study, the study coordinator (SB) will create an electronic folder with a pseudonym for each participating kindergarten on a secure server. Documentation files with the following data will be stored in this folder:

– Main target parameters (missed days because of an infection)
– Secondary target parameters (CCQ)
– Interview audio and transcripts
– Interview and observation field notes.

All results will be analyzed through anonymous aggregated data (quantitative part) or with the use of pseudonyms (qualitative part).

All collected study data will be collected and handled conforming to the Berlin Data protection Law (Berliner Datenschutzgesetz – BlnDSG) and in adherence with the Declaration of Helsinki code of ethics. The files will be stored in their anonymized and/or pseudonymized form after completion of the study on a Charité server for 10 years. Within the electronic survey system (SoSci), the participants are encrypted using IDs and are therefore not identifiable. The study coordinator (SB) will store the encryption list and will keep it in a locked cabinet. Access to the collected data will be restricted to members of the study team (SB, AT, MG, MB, GS, SW).

The onboarding and weekly questionnaires will be sent using the collected parent emails using the Charité version of the software SoSci (Program-Version 3.5.00).

After an initial invitation, caregivers who have not completed the survey will be reminded to complete it on a weekly basis over 6 weeks. Additionally, a study assistant (AT) will contact caregivers of missing entries via telephone. In addition to the online entries, the missed kindergarten days will be documented by the kindergarten facilities and likewise measured at the two time points for 6 weeks at baseline and 12 months after baseline.

The total number of kindergarten educator sick days will also be documented without descriptive personal data and kindergartens will report the used interventions weekly to the study assistant.

### 2.8 Statistical methods

The study will be published in a peer-reviewed academic journal. Analyses of data will be performed after the collection is finalized according to the following hypotheses:

- Primary Efficacy Hypothesis (Infection days leading to absence):
- Primary Null Hypothesis (H0): Implementation of a child-friendly Kneipp concept in the kindergartens does not lead to a significant reduction in infection-related absenteeism, after 12 months.
- Alternative Hypothesis (H1): Implementation of child-friendly Kneipp concept in the kindergartens results in a significant reduction in infection-related absenteeism, after 12 months.

Participant characteristics, stratified by groups (Kneipp vs. Control) will be presented. For continuous variables, descriptive statistics such as mean, standard deviation, and range will be calculated, while frequencies and percentages will be used for categorical variables.

The primary outcome will be the number of sick days reported to the kindergarten. To account for the expected overdispersion (right-skewed data because of high zero counts) in the count data, we will employ a negative binomial regression model. This model will include the group (Kneipp vs. Control) and the number of sick days at baseline as predictors. We will integrate unique kindergarten identifiers to determine if cluster effects exist.

For secondary outcome analysis, we will conduct an independent t-tests to compare the mean Common Cold Questionnaire (CCQ) scores between the Kneipp and Control groups. To investigate the interaction effects of group and sex, and group and age on CCQ scores, a two-way ANOVA will be performed. This model will include the main effects of group, sex, and age, as well as their interactions. If significant interactions are identified, post-hoc analyses will be conducted. Correlation analysis will involve calculating Spearman’s correlation coefficients to assess the relationships between CCQ scores and the number of sick days, and between CCQ scores and age. The correlation (ρ) will be interpreted based on conventional guidelines. To compare the frequencies of health outcomes such as colds, bronchitis, lung inflammation, antibiotic use, and gastrointestinal infections between the Kneipp and Control groups, chi-square tests will be performed. If the assumptions of the chi-square test are violated due to expected frequencies being less than 5, Fisher’s exact test will be used instead.

The compliance analysis will be conducted by considering both the Intention-to-Treat (ITT) and Per-Protocol (PP) populations. The ITT population will include all children from whom kindergarten reports are available. The PP population will consist of children who strictly followed the prescribed treatment and for whom complete data is available.

For handling missing data in the ITT approach, multiple imputation will be used. Sensitivity analyses will also be performed to compare the results from the ITT and PP approaches to ensure robustness of the findings.

To ensure the validity of our model assumptions, normality of the data distribution for continuous outcomes will be assessed using the Shapiro–Wilk test. If necessary, data will be transformed using appropriate methods, such as log transformation, to meet the assumptions on the statistical tests. All analysis will be considered significant if *p* < 0.05 and will be conducted using R in its current version.

Qualitative data, focused ethnography observation protocols and interview transcripts, will be analyzed with content analysis with a combined deductive and inductive analysis (18).

### 2.9 Monitoring

Regular site visits will be done in all kindergarten facilities by the study team (SB, MG, AT) to insure the complete and secure documentation of the study data. These will be kept in a specific folder and stored in a cabinet in a locked office.

### 2.10 Harms

The safety protocols will be created and then adapted to the kindergarten environment based on potential harms listed in the NCI-CTCAE (17) that could occur within the framework of the interventions. The forms will be discussed with experts (developer of the Kita Kneipp-intervention curriculum and a methodological expert) and linguistically adapted to laypersons’ terms.

The risks of this study are low. Nevertheless, there is a possibility the application of the Kneipp concept could place an additional time burden on educators. There is no risk for the children.

A steering committee made up of external experts for Kneipp therapy and prevention research will meet quarterly to discuss problems and monitor safety. Additionally, the quality assurance office of the Charité will be consulted on safety measures. Site visits will be planned during the duration of the study.

Adverse events (AEs) are not to be expected as a result of participation. However, should any occur, the applications can be stopped immediately by the educators. All adverse events and suspected cases will be documented by the educators or the daycare center management and reported to the study management (SB, AT, MG) within 24 h. If there are any directly reported AEs related to Kneipp, the interventions will be immediately stopped and reviewed by the quality assurance team (GS – principal investigator, SB, FK, SS – experienced biostatistician, WS – experienced clinical study coordinator). Symptoms to be recorded are: general, skin, falls, and breathing, and a three-point scale (mild, moderate or severe) will be used (see Supplementary material: Adverse Events Documentation Form).

### 2.11 Auditing

The interventions observed at two time points not only will provide data for the qualitative part but will enable the study team to observe and supervise the applications.

## 3 Discussion

To our knowledge, this planned study is the first of its kind to specifically investigate the effects of a Kneipp intervention in kindergarten facilities. As a low-cost measure with some indicated potential to improve the health of children (8, 9), this confirmatory, mixed-method, two-armed, waitlist, clinical, cluster-RCT study shows promise for presenting evidence of a Kneipp intervention within a daycare setting. A prior RCT study about hydrotherapy in children aged 3–7 years old (intervention group – hydrotherapy and saline inhalation, control group - saline inhalation), without the holistic aspect of Kneipp found no significant results regarding incidence of colds and average duration of cold episodes (19). Seeing if a broad health-promoting concept such as Kneipp would be more beneficial is of interest. In addition to the primary and secondary outcomes the qualitative documentation has the potential to not only track the effects but also to describe how the intervention is conducted with the children and perceived by the educators. That would help recognize and evaluate possible challenges and potentials of applying such a holistic approach in childcare facilities.

Several limitations to this confirmatory study design can be considered. The pre-selection based on socio-demographic information and cluster grouping of the kindergartens to be randomized may introduce some allocation bias into the randomization process. Further, the study primary and secondary outcomes rely on self-reported measures of data gathering on the part of parents and kindergarten directors. The open-label study design could influence the subjective experience of the cold and sickness symptoms and how they report or attend kindergarten. Caregivers may show some response bias, as they are informed of the purposes of the Kneipp concept. Nevertheless, the primary measure will be double recorded by caregivers and kindergarten directors to achieve a closer approximation for internal validity. Further, the CCQ relies on the recall of symptoms by caregivers that could be affected over time. For minimizing this limitation, caregivers will be prompted to fill out the form on a weekly basis and a member of the study group (AT) will monitor the responses and, if needed, a reminder e-mail will be sent.

## 4 Ethics and dissemination

### 4.1 Protocol amendments

Any changes to the protocol which may have an impact on the study conduct, potential benefit of the study group or may affect its safety, including modifications of the study objectives, design, population, sample sizes, procedures, or significant administrative changes will require a formal amendment to the protocol. Such an amendment will be approved by the Ethics Committee of Charité – Universitätsmedizin Berlin.

### 4.2 Consent or assent

A researcher from the study team will explain the trial to the directors of the kindergarten facilities. Educators and caregivers/caregivers will receive a document explaining the study purposes and a written consent form which will be collected from the educators and stored in a locked office in a secure folder in the facilities (See Supplementary materials). If there are any questions left, online meetings will be organized, where caregivers and educators will have the opportunity to talk to the project coordinator or principal investigator (SB or GS). Through regular visits in the kindergarten facilities throughout the whole study period, the same form and written consent will be collected from newly hired kindergarten staff, who will also get the chance to talk to a study researcher (SB or MG) and be informed of the study plan and purposes. Furthermore, information brochures about the used Kneipp interventions will be distributed in the kindergartens, providing additional information for the children’s caregivers on the used applications. Illustrated cards with the interventions will be used to explain the method in a child friendly way to the study population.

### 4.3 Confidentiality

Personal information about the children, their caregivers, and the educators will be securely stored in a locked office in the facilities. The interviews done with the educators will be auto transcribed and corrected to pseudonymize any personal information.

### 4.4 Declaration of interest

The study is being financially supported by a German health insurance company – ‘MKK – Meine Krankenkasse’.

### 4.5 Access to data

Upon completion of the study, all anonymized data, custom written codes, and comprehensive documentation will be deposited in a dedicated online repository. The repository will be public, allowing open access to the dataset.

### 4.6 Ancillary and post-trial care

No follow-up services are provided to participants after their involvement in the trial has ended.

### 4.7 Dissemination policy

Two publications with the results (one on the quantitative and one on the qualitative part) is planned to be submitted in a peer-reviewed academic journal and made public to other researchers, participants etc. At the end of the study (November 2024) participants – parents and educators, researchers from the study group, the sponsor and Kneipp-Bund e.V. members will additionally gather to discuss the results.

## Author contributions

MG: Investigation, Visualization, Writing – original draft, Writing – review & editing, Formal analysis, Project administration, Data curation. SS: Data curation, Formal analysis, Methodology, Validation, Writing – review & editing. MB: Software, Writing – review & editing, Investigation. FK: Methodology, Formal Analysis, Validation, Data curation, Writing – review & editing. AT: Data curation, Investigation, Project administration, Writing – review & editing. MJ: Conceptualization, Methodology, Writing – review & editing, Funding acquisition. WS: Conceptualization, Methodology, Supervision, Validation, Writing – review & editing. SB: Data curation, Formal analysis, Investigation, Methodology, Project administration, Supervision, Validation, Writing – original draft, Writing – review & editing, Visualization. GS: Conceptualization, Funding acquisition, Methodology, Resources, Supervision, Validation, Writing – review & editing.
