# Correction: Effect of the health and wellness Kneipp concept on health promotion and reduction of sick days for kindergarten children: a cluster randomized controlled trial protocol

**DOI:** 10.3389/fmed.2026.1866546

**Published:** 2026-05-13

**Authors:** Marinela Gerganova, Steven Schepanski, Martin Bogdanski, Farid I. Kandil, Angela Tekath, Michael Jeitler, Wiebke Stritter, Sarah B. Blakeslee, Georg Seifert

**Affiliations:** 1 Charité Competence Center for Traditional and Integrative Medicine (CCCTIM), Charité – Universitätsmedizin Berlin, corporate member of Freie Universität Berlin and Humboldt – Universität zu Berlin, Berlin, Germany; 2 Department of Pediatrics, Division of Oncology and Hematology, Charité – Universitätsmedizin Berlin, corporate member of Freie Universität Berlin and Humboldt – Universität zu Berlin, Berlin, Germany; 3 Institute of Social Medicine, Epidemiology and Health Economics, Charité – Universitätsmedizin Berlin, corporate member of Freie Universität Berlin and Humboldt – Universität zu Berlin, Berlin, Germany; 4 Department of Internal Medicine and Nature-Based Therapies, Immanuel Hospital Berlin, Berlin, Germany

**Keywords:** Kneipp, child health promotion, salutogenesis, kindergarten, hydrotherapy, lifestyle wellness, integrative medicine, common cold questionnaire

A correction has been made to section **2. Methods**, Paragraph Number 1. The correct text appears below:

“The Kita Kneipp Study is a confirmatory, mixed-method, two-armed, waitlist, clinical, cluster RCT that was registered with the German Clinical Trial Register (DRKS-ID: DRKS00029275) on November 10, 2022 (submission for registration June 22, 2022), and approved by the Ethics Committee of the Charité - Universitätsmedizin Berlin (Application number: EA2/122/22, approved on September 13, 2022). It follows the standards of the Helsinki convention of good clinical practices.”

The original version of this article has been updated.

