# Supplementary material for: Effect of the health and wellness Kneipp concept on health promotion and reduction of sick days for kindergarten children: a cluster randomized controlled trial protocol
Source: Front Med (Lausanne). 2024 Jul 26;11:1412971. doi: 10.3389/fmed.2024.1412971 (PMC11309992; doi:10.3389/fmed.2024.1412971)
Supplement: Supplementary file 2 [file Data_Sheet_2.PDF]

CHARITÉ

ChariteCentrum für Frauen-, Kinder- und Jugendmedizin mit Perinatalzentrum und Humangenetik

Charité | Campus Virchow-Klinikum | 13344 Berlin

Informed Consent Parents
Version 3 vom 20. September 2022

Klinik für Pädiatrie m. S. Onkologie/Hämatologie/SZT
Direktorin Prof. Dr. med. Angelika Eggert
Stellvertretender Direktor Prof. Dr. med. Dr. Karl-Heinz Seeger
Sekretariat Belgin Kömür
Tel. +49 30 450 566 132
Fax +49 30 450 566 906
Hausanschrift
Charité-CVK, Augustenburger Platz 1, 13353 Berlin (Wedding)
http://paedonko.charite.de/

AG Integrative Medizin in der pädiatrischen Onkologie
Leitung Prof. Dr. med. Georg Seifert
Tel. + 49 30 450 566 058
Fax + 49 30 450 566 965

Integration of Kneipp hydrotherapy for children in daycare centers - a model project for health prevention in Berlin daycare centers with scientific evaluation

I (first name, surname) ____, as parent/legal guardian of the child (first name, surname) ______________________, born on ______________ (date of birth dd/mm/yyyy), hereby declare that I have been informed in writing about the nature and significance of the above-mentioned study in accordance with the study information version 3 dated September 20, 2022. All my questions have been answered to my satisfaction. I declare my consent to participate in the above-mentioned study.

For study purposes (sending questionnaires) I can be contacted via the following e-mail address and telephone number (please write legibly in block letters below)

Telephone number: ____

E-Mail:

☐☐☐☐☐☐☐☐☐☐☐☐☐☐☐☐☐☐☐☐☐☐☐☐@☐☐

☐☐☐☐☐☐☐☐☐☐☐☐☐☐☐☐☐☐

Information and consent to data processing

I am aware that I have the right to withdraw my consent at any time without giving reasons and without adverse consequences for me and that I can object to further processing of my data and samples and request their destruction.

I have received a copy of the written study information and the declaration of consent with version date 20.09.2022.

I agree that in the context of the above-mentioned study:

1. my personal data necessary for the purpose of the above-mentioned study will be collected by the study coordinator* as described in the study information of version 3 dated 20.09.2022 and recorded and processed in pseudonymized form, including on electronic data carriers;

2. that the study results will be published in an anonymous form that does not allow any conclusions to be drawn about my person.

Place…………………………………. Date……………………………………. Signature…………………………………….

Confirmation of the informing study staff member

The study participant was informed in writing about the above-mentioned study and had the opportunity to ask questions about the study. A copy of the study information and the informed consent form was given to him/her.

Place…………………………………. Date……………………………………. Signature…………………………………….
