# Supplementary material for: Effect of the health and wellness Kneipp concept on health promotion and reduction of sick days for kindergarten children: a cluster randomized controlled trial protocol
Source: Front Med (Lausanne). 2024 Jul 26;11:1412971. doi: 10.3389/fmed.2024.1412971 (PMC11309992; doi:10.3389/fmed.2024.1412971)
Supplement: Supplementary file 3 [file Data_Sheet_3.PDF]

Study Information Educators

Version 3 dated September 20, 2022

Integration of Kneipp's Hydrotherapy for Children in Daycare Centers - A Model Project for Health Prevention in Berlin's Daycares with Scientific Evaluation

Dear study participant,

With the aforementioned project, we aim to investigate the health-promoting effects of Kneipp water applications (hydrotherapy) in Berlin's daycare centers. The water applications according to Kneipp stem from a long-standing naturopathic tradition of Kneipp's health teachings and health promotion, which are based on five pillars. These include hydrotherapy, herbal medicine, movement, nutrition, and lifestyle rhythm. The five pillars are already in alignment with many aspects of the Berlin daycare educational program. This concept has been successfully practiced for over 15 years in Kneipp-certified daycares as a means of health promotion.

As part of a scientific evaluation of the certification process, we want to examine to what extent a Kneipp concept can lead to fewer illnesses among daycare children in Berlin.

Your daycare wishes to obtain certification as a Kneipp kindergarten through the Kneipp Association and participate in the evaluation.

Procedure and Duration of Participation

For this, we need your support as educators during the project period from the beginning (autumn 2022) to the end (autumn 2023).

If you wish to participate in the study and sign the consent form, you will be asked at the beginning of the study (autumn 2022) to document the daycare absences and the reasons for the absences (illness/no illness) of the daycare children using a provided documentation sheet.

Starting from November 2022 (or November 2023 if your daycare is in the control group).

As part of the certification, you (and/or your colleagues, in consultation with your management) will be able to participate in a 4-day training course to become a Kneipp educator with the Kneipp Association. This training will enable you (and/or your colleagues)

to implement the Kneipp concept of the Kneipp Association in daycare everyday life. The training and implementation of the Kneipp concept are based on the guidelines of the Kneipp Association and will be individually adapted to your team and the circumstances of your daycare.

Data collection as part of the accompanying evaluation of the study will largely be through parents. Parents who wish to participate in the study and sign the consent form will receive from us at the beginning of the study (from autumn 2022) and at the end of the study (autumn 2023) a digital link to a short questionnaire once a week by email for a period of 2 months. In this questionnaire, parents will be asked to indicate whether and if so, how long their child was ill in the past week.

As part of the evaluation, we will ask you (and/or your colleagues) to document the children's absences over 2 separate 6-week evaluation periods (autumn 2022 and 2023). This documentation will be entered on a pre-prepared form and then passed on to the study coordinator.

At the end of the study, a study staff member will visit the daycare on two occasions to observe how the Kneipp concept is implemented in the daycare and whether any changes in interaction between educators and children can be observed. The recorded observations will be collected and analyzed without any personal data.

Furthermore, (voluntary) interviews will be conducted with educators or directors. If you are selected and agree to participate in the interview, we will conduct a 30-45 minute interview about your experiences with the Kneipp concept (either live, by phone, or video conference software, as agreed upon). These interviews will be recorded via audio, transcribed without personal data, and scientifically evaluated.

Voluntary Participation

Participation in data collection as part of the accompanying evaluation is voluntary and independent of your daycare's certification by the Kneipp Association and your (potential) training as a Kneipp educator. There will be no disadvantages to you if you do not agree to the data collection.

You have the right to withdraw your consent to participate in the study or to further process your data at any time and to end your participation in the study.

Risks of Participation

The study evaluates the implementation of the Kneipp concept according to the guidelines of the Kneipp Association. Your daycare wishes to be certified as a Kneipp daycare according to the guidelines of the Kneipp Association and to implement the Kneipp concept in the daycare. The implementation of the concept and the four-day training may lead to additional time and effort. No further risks of participation are expected.

Insurance Coverage

No special insurance has been taken out for participants in this study. The staff involved in the study at Charité - Universitätsmedizin Berlin are insured against liability claims resulting from their negligent behavior by Charité's liability insurance.

Data Protection - What Happens to Your Data?

The processing of your personal data is based on the EU General Data Protection Regulation and the Berlin Data Protection Act.

The collection and processing of personal data (e.g., contact details for an interview) as well as study data are carried out exclusively for the purpose of the aforementioned study and in accordance with the relevant legal regulations, in particular the General Data Protection Regulation (GDPR) and the Berlin Data Protection Act (BDSG). The collection and processing of personal data are only lawful with your consent (Article 6 GDPR, § 51 BDSG-new).

By signing the consent form, you agree that the study management and study staff may collect and process your pseudonymized data presented in the participant information for the purpose of the aforementioned study. Except for reactions to adverse events and for the most important steps of the study logistics, your personal data will be processed exclusively in pseudonymized form, meaning that your name will be replaced by a code (e.g., B022). The study leader and study coordinator are responsible for data processing in accordance with the EU General Data Protection Regulation.

Already at the beginning of the study, with the consent form, a code (pseudonym) will be assigned to you as a participant in this study and also to the participating children, under which the storage and evaluation of the data collected in the study will take place. The signed consent forms and all personal data (names, dates of birth, email addresses, telephone

numbers, and postal addresses) remain securely stored in the study center of the Integrative Medicine in Pediatric Oncology at Charité - Universitätsmedizin Berlin. Only the study leader and study coordinator have access to decryption; for all other persons, traceability is not possible. Your data will be treated with care and used only for absolutely necessary study purposes. They will not be published or passed on to third parties. All data are pseudonymously stored on specially secured servers at Charité. They are safe from theft and will not be passed on to third parties not mentioned in this statement.

The study coordinator will pass on the study-related pseudonymized data collected during the study to the responsible study leader Prof. Dr. Georg Seifert and his staff for all matters concerning the practical implementation of the study and the statistical evaluation.

Audio files with personal information will be processed exclusively by the study coordinator by transcribing them without personal information and then analyzing them in a pseudonymized form. The data present at the mentioned points will be pseudonymized and processed and stored for a period of 10 years and then destroyed.

Your Rights as Participants

In connection with the personal data processed as part of this study, you have the following rights:

You can withdraw your consent to the processing of the data at any time. To do so, please contact the study leader and/or study coordinator (sarah.blakeslee@charite.de). Please note that the legality of the data processing carried out up to that point will not be affected, Article 7 (3) GDPR.

Right to Information: You have the right to information about the personal data concerning you that are collected and processed as part of this study (Article 15 GDPR, §§34 BDSG-new), if this is technically possible.

Right to Rectification: You have the right to have incorrect personal data concerning you corrected (Article 13 GDPR), if this is technically possible.

Right to Erasure: You have the right to have personal data concerning you erased, e.g., if this data is no longer necessary for the purpose for which it was collected (Article 17 GDPR, §§ 35 BDSG-new), if this is technically possible.

Right to Restriction of Processing: Under certain conditions, you have the right to demand the restriction of processing, i.e., the data may only be stored but not processed, if this is technically possible. You must apply for this. Please contact the study leader, study physician, or the data protection officer of the Institute for Social Medicine, Epidemiology and Health Economics, Charité - Universitätsmedizin Berlin for this purpose (Article 18 GDPR).

Right to Object: You have the right to object at any time to specific decisions or measures regarding the processing of personal data concerning you (Article 21 GDPR, § 36 BDSG-new). Such processing will then no longer take place, if this is technically possible.

Deletion of Personal Data:

The personal data will be deleted or destroyed at the latest 10 years after the end of the study.

Publication of Study Results:

The publication of study results will be in anonymized form. Anonymized means that the published results do not allow conclusions to be drawn about your identity or can only be drawn with an disproportionately large amount of time, cost, and effort. We ask for your consent for this step of anonymization, as it constitutes processing within the meaning of Article 4 (2) GDPR. Since after anonymization it is no longer possible to establish a reference to your person, the right to withdraw, information, correction, or deletion is no longer feasible.

If the responsible person for the study-related collection and processing of personal data cannot help you further, you also have the option of contacting the Data Protection Office of Charité - Universitätsmedizin Berlin:

Information on the Processing of Your Personal Data:

The signed consent forms and all personal data (names, dates of birth, email addresses, telephone numbers, and postal addresses) remain securely stored in Integrative Medicine in Pediatric Oncology at Charité - Universitätsmedizin Berlin. Only the study leader and the study coordinator have access to decryption. For all other persons, traceability is not possible. Your data will be treated with care and used only for absolutely necessary study purposes. They will not be published or passed on to third parties.

By signing the consent form, you agree to participate in the study voluntarily. You can withdraw your consent to participate in the study at any time, even if the daycare continues to participate in the study and the daycare Kneipp concept continues to be implemented. The data from the questionnaires and observation protocols will be stored in the study center of the Integrative Medicine working group in the Department of Pediatrics with a focus on oncology/hematology, Charité - Universitätsmedizin Berlin, and evaluated by the study team using statistical and qualitative methods.

The results of the study will be published.

If you have further questions about the study, please feel free to contact the study leader:

Prof. Dr. Georg Seifert, Department of Pediatrics with a focus on Hematology/Oncology/BMT, Charité - Universitätsmedizin Berlin

Charitéplatz 1

10117 Berlin

Telephone: 030/450-DW

Email: georg.seifert@charite.de

For concerns regarding data processing and compliance with data protection requirements, you can also contact the Data Protection Office of Charité - Universitätsmedizin Berlin:

Data Protection Office of Charité - Universitätsmedizin Berlin

Charitéplatz 1

10117 Berlin

Telephone: +49 30 450 580016

Email: datenschutz@charite.de

In the event that you consider data processing to be unlawful, you have the option of lodging a complaint with the supervisory authority responsible for Charité - Universitätsmedizin Berlin:

Berlin Commissioner for Data Protection and Freedom of Information

Friedrichstraße 219

10969 Berlin

We would be delighted to welcome you as a participant in our study!

With warm regards,

Your study team:

Sarah Blakeslee & Georg Seifert
