# Supplementary material for: Effect of the health and wellness Kneipp concept on health promotion and reduction of sick days for kindergarten children: a cluster randomized controlled trial protocol
Source: Front Med (Lausanne). 2024 Jul 26;11:1412971. doi: 10.3389/fmed.2024.1412971 (PMC11309992; doi:10.3389/fmed.2024.1412971)
Supplement: Supplementary file 4 [file Data_Sheet_4.PDF]

Study Information for Parents

Version 3 dated September 20, 2022

Integration of Kneipp Hydrotherapy for Children in Daycare Centers - A Model Project for Health Prevention in Berlin's Daycares with Scientific Evaluation

Dear Parents,

With the project mentioned above, we aim to evaluate the health-promoting effects of Kneipp water applications (hydrotherapy) in Berlin's daycare centers. Your daycare wishes to participate in our project and thus obtain certification as a Kneipp kindergarten through the Kneipp Association.

As part of a scientific evaluation of the certification process, we aim to investigate to what extent a Kneipp concept can lead to fewer illnesses among children in Berlin daycares.

For this, we need your support as parents: We kindly ask you to fill out some questionnaires online at the beginning (autumn 2022) and end (autumn 2023) of the project year regarding whether and how long your child was ill during this time.

First, however, we will introduce the project in its basic features so that you have a basis for decision-making:

Background, Objectives, and Benefits of the Study

In Germany, naturopathy has a long tradition. The pastor Sebastian Kneipp (1821-1897) is considered one of the younger founders of naturopathic medicine and health promotion. Water therapy, herbal medicine, movement, nutrition, and lifestyle rhythm are among the five pillars of Kneipp's health teachings. The five pillars are already in alignment with many aspects of the Berlin daycare educational program. This concept has been successfully practiced for over 15 years in Kneipp-certified daycares as a means of health promotion.

Kneipp is primarily associated with water applications and the so-called "Kneipp cure" today. The most well-known water treatments (e.g., arm douches, face douches) and wraps are also applied to children. However, there are few studies to date that examine the specific health benefits of the Kneipp approach in daycare settings.

Procedure and Duration of Participation

If you wish to participate in the study and sign the consent form, you will receive from us at the beginning of the study (from autumn 2022) and at the end of the study (autumn 2023) a digital link to a short questionnaire once a week by email for a period of 2 months. In this questionnaire, you will be asked to indicate whether and, if so, how long your child was ill in the past week. If you have not completed the questionnaires, you will receive a reminder on the same day.

You will enter your email address and your child's name and date of birth once on the consent form. We will send you a personalized link to the questionnaire via our survey system to the email address provided. The consent form with your personal data will be securely locked away and accessible only to the personnel of the Kneipp daycare study. No

further personal data (name, address, etc.) will be collected. A personally created study code for your child will be assigned when consenting to participate in the study, linking your child's data between the daycare absences entries and your information.

At the end of the study, a study staff member will visit the daycare on two occasions to observe how the Kneipp concept is implemented in the daycare and whether changes in interaction between educators and children can be observed. Again, the recorded observations will be collected and analyzed without any personal data.

Voluntary Participation

Participation in data collection as part of the accompanying evaluation is voluntary and independent of the certification of the daycare by the Kneipp Association. There will be no disadvantages to you if you do not agree to participate in the data collection.

You have the right to withdraw your consent to participate in the study or to further process your data at any time and to end your participation in the study.

Risks of Participation

Your daycare wishes to certify as a Kneipp daycare according to the guidelines of the Kneipp Association and will implement this concept in the daycare. Children may react differently to new information and routines. They will be gently introduced to the Kneipp concept according to the guidelines of the Kneipp Association. Nevertheless, it is possible that your child may not want to participate in an activity. In this case, your child is of course not obliged to participate. No further risks of participation are expected. If your child does not participate in the Kneipp application, no further evaluation data will be collected.

Insurance Coverage

No special insurance has been taken out for participants in this study. The staff involved in the study at Charité - Universitätsmedizin Berlin are insured against liability claims resulting from their negligent behavior by Charité's liability insurance.

Data Protection - What Happens to Your Data or Your Child's Data?

The processing of your personal data is based on the EU General Data Protection Regulation and the Berlin Data Protection Act.

The collection and processing of personal data as well as study data are carried out exclusively for the purpose of the aforementioned study and in accordance with the relevant legal regulations, in particular the General Data Protection Regulation (GDPR) and the Berlin Data Protection Act (BDSG). The collection and processing of personal data are only lawful with your consent (Article 6 GDPR, § 51 BDSG-new).

By signing the consent form, you agree that the study management and study staff may collect and process your pseudonymized data presented in the participant information for the purpose of the aforementioned study. Except for reactions to adverse events and for the most important steps of the study logistics, your personal data will be processed exclusively in pseudonymized form, meaning that your name will be replaced by a code (e.g., B022). The

study leader and study coordinator are responsible for data processing in accordance with the EU General Data Protection Regulation.

Already at the beginning of the study, with the consent form, a code (pseudonym) will be assigned to you as a participant in this study and also to the participating children, under which the storage and evaluation of the data collected in the study will take place.

The signed consent forms and all personal data (names and birth dates of the child, email address) will remain securely stored at Integrative Medicine in Pediatric Oncology at Charité - Universitätsmedizin Berlin. Only the study leader and study coordinator have access to decryption. For all other persons, traceability is not possible. Your data will be treated with care and used only for absolutely necessary study purposes. They will not be published or passed on to third parties. All data will be pseudonymized and stored on specially secured servers at Charité. They are safe from theft and will not be passed on to third parties not mentioned in this statement.

The study coordinator will pass on the study-related pseudonymized data collected during the study to the responsible study leader Prof. Dr. Georg Seifert and his staff for all matters concerning the practical implementation of the study and the statistical evaluation.

In addition to the information you provide in the electronic questionnaire, the daycare absences will be documented by the educators on our daycare questionnaire. The data at the mentioned points will be stored and processed in pseudonymized form and will be stored for a period of 10 years and then destroyed.

Your Rights as Participants

You have the following rights in connection with the personal data processed as part of this study:

You can withdraw your consent to the processing of the data at any time. To do so, please contact the study leader and/or study coordinator Please note that the legality of the data processing carried out up to that point will not be affected.

Right to Information: You have the right to information about the personal data concerning you that are collected and processed as part of this study, if this is technically possible.

Right to Rectification: You have the right to have incorrect personal data concerning you corrected, if this is technically possible.

Right to Erasure: You have the right to have personal data concerning you erased, e.g., if this data is no longer necessary for the purpose for which it was collected, if this is technically possible.

Right to Restriction of Processing: Under certain conditions, you have the right to demand the restriction of processing, i.e., the data may only be stored but not processed, if this is technically possible. You must apply for this.

Right to Object: You have the right to object at any time to specific decisions or measures regarding the processing of personal data concerning you. Such processing will then no longer take place, if this is technically possible.

Deletion of Personal Data:

The personal data will be deleted or destroyed at the latest 10 years after the end of the study.

Publication of Study Results:

The publication of study results will be in anonymized form. Anonymized means that the published results do not allow conclusions to be drawn about your identity or can only be drawn with a disproportionately large amount of time, cost, and effort.

If the responsible person for the study-related collection and processing of personal data cannot help you further, you also have the option of contacting the Data Protection Office of Charité - Universitätsmedizin Berlin.

Regardless of your right to take legal action, you have the right to lodge a complaint with a supervisory authority if you believe that the processing of your data is not permissible under data protection law.

Berlin Commissioner for Data Protection and Freedom of Information:

Friedrichstr. 219, 10969 Berlin

Email: mailbox@datenschutz-berlin.de

Tel.: 030 13889-0

Involved Persons:

Study Leader:

Prof. Dr. med. Georg Seifert, Charité - Universitätsmedizin Berlin, Department of Pediatrics with a focus on Oncology/Hematology/BMT, Integrative Medicine in Pediatric Oncology

Project Coordination:

Dr. rer. medic. Sarah Blakeslee, Charité - Universitätsmedizin Berlin, Department of Pediatrics with a focus on Oncology/Hematology/BMT, Integrative Medicine in Pediatric Oncology

We would be very pleased to welcome you as a participant in our study!

With warm regards,

Your Study Team:

Sarah Blakeslee & Georg Seifert
