# Supplementary material for: Effect of the health and wellness Kneipp concept on health promotion and reduction of sick days for kindergarten children: a cluster randomized controlled trial protocol
Source: Front Med (Lausanne). 2024 Jul 26;11:1412971. doi: 10.3389/fmed.2024.1412971 (PMC11309992; doi:10.3389/fmed.2024.1412971)
Supplement: Supplementary file 5 [file Data_Sheet_5.PDF]

Prompts and Answers

(Chat GPT – 3.5)

Prompt:

Can you write (in bullet points) the most important things to the 5 elements of the Kneipp concept (Sebastian Kneipp)?

AI generated answer:

‘’Certainly! Sebastian Kneipp's concept focuses on five elements that contribute to a healthy lifestyle. Here are the key points for each element:

1. **Water:**

   - Emphasizes hydrotherapy, using water in various forms for healing.

   - Cold water applications to stimulate circulation and strengthen the immune system.

   - Contrast baths (alternating hot and cold water) to promote blood flow and vitality.

   - Water treatments like water treading, arm baths, and wet compresses for specific health benefits.

2. **Plants:**

   - Advocates the use of medicinal plants and herbs for natural healing.

   - Herbal teas and infusions to support various bodily functions.

   - Recognition of the therapeutic properties of specific plants for treating ailments.

   - Promotes a balanced and plant-based diet for overall well-being.

3. **Exercise:**

   - Encourages regular physical activity for health and vitality.

   - Emphasis on outdoor activities, such as walking and hiking in natural surroundings.

   - Simple exercises to improve flexibility, strength, and overall fitness.

   - Tailoring exercise routines to individual needs and abilities.

4. **Nutrition:**

   - Stresses the importance of a balanced and wholesome diet.

   - Emphasis on whole, unprocessed foods rich in nutrients.

   - Individualized dietary recommendations based on health conditions and needs.

   - Avoidance of excessive consumption of stimulants, processed foods, and unhealthy fats.

5. **Balancing Life:**

- Recognizes the interconnectedness of physical, mental, and emotional well-being.
- Encourages stress management and relaxation techniques.
- Adequate rest and sleep as essential for the body's healing and regeneration.
- Mindfulness practices to foster a positive mindset and emotional balance.

By integrating these five elements into daily life, the Kneipp concept aims to promote holistic health and well-being.''

Prompt:

Can you translate this in English:
Studieninformation Erzieher*innen

Version 3 vom 20. September 2022
Integration von Kneippscher Hydrotherapie bei Kindern in Kindertagesstätten - ein Modellprojekt zur Gesundheitsprävention in Berliner Kitas mit wissenschaftlicher Evaluation

Liebe Studieninteressentin, lieber Studieninteressent,
mit dem oben genannten Projekt möchten wir die gesundheitsfördernden Auswirkungen von Kneippschen Wasseranwendungen (Hydrotherapie) in Berliner Kitas (Kindertagesstätte) untersuchen. Die Wasseranwendungen nach Kneipp entstammen einer langjährigen naturheilkundlichen Tradition der Kneippschen Gesundheitslehre und Gesundheitsförderung, welche auf fünf Säulen beruht. Dazu gehören die Wasserheilkunde, Kräutermedizin, Bewegung, Ernährung und der Lebensrhythmus. Die fünf Säulen sind in viele Aspekte bereits mit dem Berliner Kita Bildungsprogramm im Einklang. In vom Kneipp-Bund e.V. zertifizierten Kitas wird dieses Konzept bereits seit über 15 Jahre erfolgreich als Gesundheitsförderung praktiziert.
Im Rahmen einer wissenschaftlichen Evaluation des Zertifizierungsprozesses möchten wir untersuchen, inwiefern ein Kneipp Konzept dazu führen kann, dass Berliner Kita Kinder weniger krank werden.
Ihre Kita möchte die Zertifizierung als Kneipp Kindergarten durch den Kneipp-Bund e.V. erlangen und damit auch an der Evaluation teilnehmen.

Ablauf und Dauer der Teilnahme
Dafür brauchen wir Ihre Unterstützung als Erzieher*innen in der Projektzeit von Anfang (Herbst 2022) bis zu Ende (Herbst 2023).

Wenn Sie an der Studie teilnehmen möchten und die Einverständniserklärung unterzeichnen, werden Sie gebeten zu Beginn der Studie (Herbst 2022), die Kita Fehltage und den Grund des Fehlens (Infekt/ kein Infekt) der Kitakinder anhand eines vorgegebenen Dokumentationsbogens zu dokumentieren.
Ab November 2022 (oder November 2023, wenn Ihre Kita in der Kontrollgruppe ist).

Im Rahmen der Zertifizierung werden Sie (und/ oder Ihre Kolleg*innen, in Absprache mit Ihrer Leitung) an einer 4-tägigen Ausbildung zur Kneipp Erzieher*in des Kneipp-Bunds e.V. teilnehmen können. Durch diese Ausbildung werden Sie (und/ oder Ihre Kolleg*innen) dazu befähigt werden, das Kita Kneipp Konzept der Kneipp Bunds e.V. im Kita Alltag umzusetzen. Die Ausbildung und Umsetzung des Kneipp Konzepts orientiert sich an den Leitlinien des Kneipp-Bund e.V. und wird individuell an Ihr Team und die Gegebenheiten Ihrer Kita angepasst.
Die Datenerhebung im Rahmen der begleitenden Evaluation der Studie erfolgt weitestgehend über die Eltern. Eltern, die an der Studie teilnehmen möchten und die Einverständniserklärung unterzeichnen, erhalten von uns zu Beginn der Studie (ab Herbst 2022) und zum Ende der Studie (Herbst 2023) jeweils für den Zeitraum von 2 Monaten einmal pro Woche per E-Mail einen digitalen Link zu einem kurzen Fragebogen. In diesem Fragebogen werden die Eltern gebeten anzugeben, ob und wenn ja, wie lange ihr Kind in der vergangenen Woche krank war.
Im Rahmen der Evaluation werden wir Sie (und/ oder Ihre Kolleg*innen) auffordern, die Fehltage der Kinder über 2 separaten 6-wöchige Evaluationszeiträume (Herbst 2022 und 2023) zu dokumentieren. Diese Dokumentation wird in einem vorgefertigten Bogen eingetragen und dann die Studien-Koordinatorin weitergegeben.
Am Ende der Studie wird an zwei Terminen eine Studienmitarbeiter*in in die Kita kommen, um zu beobachten, wie das Kneipp Konzept in der Kita umgesetzt wird und ob sich Veränderungen in der Interaktion zwischen die Erzieher*innen und Kinder feststellen lassen. Die protokollierten Beobachtungen werden komplett ohne persönliche Daten erhoben und analysiert.
Des Weiteren werden (freiwillige) Interviews mit Erzieher*innen oder Leiter*innen durchgeführt. Falls Sie ausgewählt werden und der Teilnahme am Interview zustimmen, werden wir ein ca. 30-45 Min. langes Interview zu Ihren Erfahrungen mit dem Kneipp Konzept mit Ihnen führen (nach Absprache entweder live, per Telefon oder Videokonferenzsoftware). Diese Interviews werden über eine Audiogerät aufgenommen, ohne personenbezogene Daten transkribiert und wissenschaftlich ausgewertet.

Freiwilligkeit der Teilnahme

Die Teilnahme an der Datenerhebung im Rahmen der begleitenden Evaluation ist freiwillig und damit unabhängig von der Zertifizierung Ihrer Kita durch den Kneipp-Bund e.V. sowie Ihrer (eventuellen) Ausbildung als Kneipp Erzieher*in. Es entstehen Ihnen diesbezüglich keine Nachteile, sollten Sie der Datenerhebung nicht zustimmen.
Sie haben jederzeit das Recht, Ihre gegebene Einwilligung zur Studienteilnahme oder zur Weiterverarbeitung Ihrer Daten zu widerrufen und die Studienteilnahme zu beenden.

Risiken der Teilnahme

Die Studie evaluiert die Umsetzung des Kneipp Konzepts nach Kneipp-Bund e.V. Leitlinien. Ihre Kita möchte sich nach den Leitlinien des Kneipp Bunds e.V. als Kneipp Kita zertifizieren lassen und das Kneipp Konzept in die Kita umsetzen. Die Umsetzung des Konzepts und die vier-tägige Ausbildung könnte zu zusätzlichem Zeit- und Arbeitsaufwand führen. Es werden keine weiteren Risiken der Teilnahme erwartet.

Versicherungsschutz

Für diese Studie wurde keine spezielle Versicherung für die Teilnehmenden abgeschlossen. Die an der Studie beteiligten Mitarbeiter*innen der Charité – Universitätsmedizin Berlin ist durch die Betriebshaftpflichtversicherung der Charité

gegen Haftpflichtansprüche, welche aus ihrem schuldhaften Verhalten resultieren könnten, versichert.

Datenschutz – Was passiert mit Ihren Daten?

Die Verarbeitung Ihrer personenbezogenen Daten erfolgt auf der Grundlage der Datenschutzgrundverordnung der EU und des Landesdatenschutzgesetzes Berlin. Die Erhebung und Verarbeitung der personenbezogenen Daten (z.B. Kontaktdaten für einem Interview) sowie der Studiendaten erfolgt ausschließlich zum Zwecke der o.g. Studie und folgt gemäß den einschlägigen rechtlichen Bestimmungen, insbesondere der Datenschutzgrundverordnung (DSGVO) und dem Berliner Datenschutzgesetz (BDSG). Die Erhebung und Verarbeitung der personenbezogenen Daten ist nur mit Ihrer Einwilligung rechtmäßig (Artikel 6 DSGVO, § 51 BDSG-neu).

Durch Ihre Unterschrift auf der Einwilligungserklärung erklären Sie sich damit einverstanden, dass die Studienleitung und die Studienmitarbeiter*innen Ihre in der Teilnahmeinformation dargestellten pseudonymisierten Daten zum Zweck der o.g. Studie erheben und verarbeiten dürfen. An allen Stellen außer zur Reaktion bei unerwünschten Ereignissen und für die wichtigsten Schritte der Studienlogistik werden Ihre persönlichen Daten ausschließlich pseudonymisiert verarbeitet, das heißt, dass Ihr Name durch einen Code (z.B. B022) ersetzt wird. Der Studienleiter und Studienkoordinatorin sind der für die Datenverarbeitung Verantwortlichen im Sinne der EU-Datenschutzgrundverordnung.

Schon mit der Einwilligungserklärung zu Beginn der Studie wird Ihnen als Teilnehmer*in dieser Studie und auch die teilnehmenden Kinder ein Code zugeordnet (Pseudonym), unter dem die Speicherung und Auswertung der in der Studie erhobenen Daten erfolgt. Die unterschriebenen Einwilligungserklärungen und alle persönlichen Daten (Namen, Geburtsdaten, E-Mail-Adressen, Telefonnummern und Postadressen) verbleiben abgeschlossen in der Studienzentrale der Arbeitsgruppe Integrative Medizin in der pädiatrischen Onkologie an der Charité – Universitätsmedizin Berlin. Nur der Studienleiter und Studienkoordinatorin haben Zugriff auf die Entschlüsselung, für alle anderen Personen ist die Rückverfolgung nicht möglich. Ihre Daten werden sorgsam behandelt und nur an zu unbedingt notwendigen Studienzwecken verwendet. Sie werden keinesfalls veröffentlicht oder an Dritte weitergegeben. Alle Daten werden pseudonymisiert auf speziell gesicherten Servern der Charité gespeichert. Sie sind damit sicher vor Diebstahl und werden keinesfalls an nicht in dieser Erklärung aufgeführte Dritte weitergegeben.

Die Studienkoordinatorin gibt die während der Studie erhobenen studienbezogenen pseudonymisierten Daten weiter an den verantwortlichen Studienleiter Prof. Dr. Georg Seifert und seine Mitarbeiter*innen für alle Angelegenheiten der praktischen Durchführung der Studie und die statistische Auswertung.

Audiodateien mit persönlichen Angaben werden ausschließlich von der Studienkoordinatorin weiterverarbeitet, indem sie ohne personenbezogene Angaben transkribiert und anschließend pseudonymisiert analysiert werden. Die bei den genannten Stellen vorhandenen Daten werden pseudonymisiert gespeichert und verarbeitet und für die Zeit von 10 Jahren gespeichert und danach vernichtet.

Ihre Rechte als Teilnehmerinnen

Sie haben im Zusammenhang mit der im Rahmen dieser Studie verarbeiteten personenbezogenen Daten folgende Rechte:

Sie können Ihre erteilte Einwilligung zur Verarbeitung der Daten jederzeit widerrufen. Nehmen Sie hierzu Kontakt mit dem Studienleiter und/oder Studienkoordinatorin (sarah.blakeslee@charite.de) auf. Beachten Sie bitte, dass die Rechtmäßigkeit der

bis dahin erfolgten Verarbeitung Ihrer Daten nicht berührt wird, Art. 7 Absatz 3 DSGVO.
Recht auf Auskunft: Sie haben das Recht auf Auskunft über die Sie betreffenden personenbezogenen Daten, die im Rahmen dieser Studie erhoben und verarbeitet werden (Artikel 15 DSGVO, §§34 BDSG-neu), sofern dies technisch möglich ist.
Recht auf Berichtigung: Sie haben das Recht, Sie betreffende unrichtige personenbezogene Daten berichtigen zu lassen (Artikel 13 DSGVO), soweit technisch möglich.
Recht auf Löschung: Sie haben das Recht auf Löschung Sie betreffender personenbezogenen Daten, z. B. wenn diese Daten für den Zweck, für den sie erhoben wurden, nicht mehr notwendig sind (Artikel 17 DSGVO, §§ 35 BDSG-neu), soweit technisch möglich.
Recht auf Einschränkung der Verarbeitung: Unter bestimmten Voraussetzungen haben Sie das Recht, die Einschränkung der Verarbeitung zu verlangen, d. h., die Daten dürfen nur gespeichert, nicht verarbeitet werden, soweit technisch möglich. Dies müssen Sie beantragen. Wenden Sie sich hierzu bitte an den Studienleiter, Studienarzt oder an den Datenschutzbeauftragten des Instituts für Sozialmedizin, Epidemiologie und Gesundheitsökonomie, Charité – Universitätsmedizin Berlin (Artikel 18 DSGVO).
Widerspruchsrecht: Sie haben das Recht, jederzeit gegen konkrete Entscheidungen oder Maßnahmen zur Verarbeitung der Sie betreffenden personenbezogenen Daten Widerspruch einzulegen (Artikel 21 DSGVO, § 36 BDSG-neu). Eine solche Verarbeitung findet anschließend nicht mehr statt, soweit technisch möglich.
Löschung personenbezogener Daten:
Die personenbezogenen Daten werden spätestens 10 Jahre nach Studienende gelöscht bzw. vernichtet.
Veröffentlichung der Studienergebnisse:
Die Veröffentlichung der Studienergebnisse erfolgt in anonymisierter Form. Anonymisiert bedeutet, dass sich aus den veröffentlichten Ergebnissen keine Rückschlüsse bzw. diese nur mit einem unverhältnismäßig großen Aufwand an Zeit, Kosten und Arbeitskraft auf die Identität Ihrer Person ableiten lassen. Für den Schritt der Anonymisierung erfragen wir Ihre Einwilligung, da es sich um eine Verarbeitung im Sinne von Art. 4 Absatz 2 DSGVO handelt. Da nach einer Anonymisierung ein Bezug zu Ihrer Person nicht mehr herstellbar ist, ist der Anspruch auf Widerruf, Auskunft, Berichtigung oder Löschung nicht mehr durchführbar.
Sollte Ihnen der Verantwortliche für die studienbedingte Erhebung und Verarbeitung personenbezogener Daten nicht weiterhelfen können, haben Sie auch die Möglichkeit mit der Stabsstelle Datenschutz der Charité Kontakt aufzunehmen:
Informationen zur Verarbeitung Ihrer personenbezogenen Daten:
Die unterschriebenen Einwilligungserklärungen und alle persönlichen Daten (Namen, Geburtsdaten, E-Mail-Adressen, Telefonnummern und Postadressen) verbleiben abgeschlossen in der Integrative Medizin in der pädiatrischen Onkologie an der Charité – Universitätsmedizin Berlin. Nur der Studienleiter und die Studienkoordinatorin hat Zugriff auf die Entschlüsselung. Für alle anderen Personen ist die Rückverfolgung nicht möglich. Ihre Daten werden sorgsam behandelt und nur zu unbedingt notwendigen Studienzwecken verwendet. Sie werden keinesfalls veröffentlicht oder an Dritte weitergegeben.
Mit der Unterzeichnung der Einwilligungserklärung erklären Sie sich einverstanden damit, an die Studie freiwillig teilzunehmen. Sie können die Einwilligung in die Studienteilnahme jederzeit zurückziehen, auch wenn die Kita weiterhin an der Studie teilnimmt und das Kita Kneipp Konzept weiterhin durchgeführt wird. Die Daten aus

den Fragebögen und Protokolle der Beobachtung werden in der Studienzentrale der AG Integrative Medizin der Klinik für Pädiatrie m. S. Onkologie/ Hämatologie, Charité – Universitätsmedizin Berlin gespeichert und vom Studienteam anhand statistischer und qualitativer Methoden ausgewertet.

Die Ergebnisse der Studie werden veröffentlicht.
Sollten Sie weitere Fragen zur Studie haben, können Sie sich gerne an die Studienleitung wenden:
Prof. Dr. Georg Seifert, Klinik für Pädiatrie m. S. Hämatologie/ Onkologie/ SZT, Charité - Universitätsmedizin Berlin

Charitéplatz 1
10117 Berlin
Telefon: 030/450-DW
E-Mail: georg.seifert@charite.de
Bei Anliegen zur Datenverarbeitung und zur Einhaltung der datenschutzrechtlichen Anforderungen können Sie sich auch an die Stabsstelle Datenschutz der Charité – Universitätsmedizin Berlin wenden:
Stabsstelle Datenschutz der Charité – Universitätsmedizin Berlin
Charitéplatz 1
10117 Berlin
Telefon: +49 30 450 580016
E-Mail: datenschutz@charite.de

Für den Fall, dass Sie eine Datenverarbeitung für rechtswidrig halten, haben Sie die Möglichkeit, bei der für die Charité - Universitätsmedizin Berlin zuständigen Aufsichtsbehörde Beschwerde einzureichen:
Berliner Beauftragte für Datenschutz und Informationsfreiheit
Friedrichstraße 219
10969 Berlin
Wir würden uns sehr freuen, Sie als Teilnehmer/ Teilnehmerin unserer Studie begrüßen zu dürfen!
Mit herzlichen Grüßen,
Ihr Studienteam:
Georg Seifert und Sarah Blakeslee

AI generated answer:

Study Information Educators

Version 3 dated September 20, 2022

Integration of Kneipp's Hydrotherapy for Children in Daycare Centers - A Model Project for Health Prevention in Berlin's Daycares with Scientific Evaluation

Dear prospective study participant,

With the aforementioned project, we aim to investigate the health-promoting effects of Kneipp water applications (hydrotherapy) in Berlin's daycare centers. The water applications according to Kneipp stem from a long-standing naturopathic tradition of Kneipp's health teachings and health promotion, which are based on five pillars. These include hydrotherapy,

herbal medicine, movement, nutrition, and lifestyle rhythm. The five pillars are already in alignment with many aspects of the Berlin daycare educational program. This concept has been successfully practiced for over 15 years in Kneipp-certified daycares as a means of health promotion.

As part of a scientific evaluation of the certification process, we want to examine to what extent a Kneipp concept can lead to fewer illnesses among daycare children in Berlin.

Your daycare wishes to obtain certification as a Kneipp kindergarten through the Kneipp Association and participate in the evaluation.

Procedure and Duration of Participation

For this, we need your support as educators during the project period from the beginning (autumn 2022) to the end (autumn 2023).

If you wish to participate in the study and sign the consent form, you will be asked at the beginning of the study (autumn 2022) to document the daycare absences and the reasons for the absences (illness/no illness) of the daycare children using a provided documentation sheet.

Starting from November 2022 (or November 2023 if your daycare is in the control group).

As part of the certification, you (and/or your colleagues, in consultation with your management) will be able to participate in a 4-day training course to become a Kneipp educator with the Kneipp Association. This training will enable you (and/or your colleagues) to implement the Kneipp concept of the Kneipp Association in daycare everyday life. The training and implementation of the Kneipp concept are based on the guidelines of the Kneipp Association and will be individually adapted to your team and the circumstances of your daycare.

Data collection as part of the accompanying evaluation of the study will largely be through parents. Parents who wish to participate in the study and sign the consent form will receive from us at the beginning of the study (from autumn 2022) and at the end of the study (autumn 2023) a digital link to a short questionnaire once a week by email for a period of 2 months. In this questionnaire, parents will be asked to indicate whether and if so, how long their child was ill in the past week.

As part of the evaluation, we will ask you (and/or your colleagues) to document the children's absences over 2 separate 6-week evaluation periods (autumn 2022 and 2023). This documentation will be entered on a pre-prepared form and then passed on to the study coordinator.

At the end of the study, a study staff member will visit the daycare on two occasions to observe how the Kneipp concept is implemented in the daycare and whether any changes in interaction between educators and children can be observed. The recorded observations will be collected and analyzed without any personal data.

Furthermore, (voluntary) interviews will be conducted with educators or directors. If you are selected and agree to participate in the interview, we will conduct a 30-45 minute interview about your experiences with the Kneipp concept (either live, by phone, or video conference

software, as agreed upon). These interviews will be recorded via audio, transcribed without personal data, and scientifically evaluated.

Voluntary Participation

Participation in data collection as part of the accompanying evaluation is voluntary and independent of your daycare's certification by the Kneipp Association and your (potential) training as a Kneipp educator. There will be no disadvantages to you if you do not agree to the data collection.

You have the right to withdraw your consent to participate in the study or to further process your data at any time and to end your participation in the study.

Risks of Participation

The study evaluates the implementation of the Kneipp concept according to the guidelines of the Kneipp Association. Your daycare wishes to be certified as a Kneipp daycare according to the guidelines of the Kneipp Association and to implement the Kneipp concept in the daycare. The implementation of the concept and the four-day training may lead to additional time and effort. No further risks of participation are expected.

Insurance Coverage

No special insurance has been taken out for participants in this study. The staff involved in the study at Charité - Universitätsmedizin Berlin are insured against liability claims resulting from their negligent behavior by Charité's liability insurance.

Data Protection - What Happens to Your Data?

The processing of your personal data is based on the EU General Data Protection Regulation and the Berlin Data Protection Act.

The collection and processing of personal data (e.g., contact details for an interview) as well as study data are carried out exclusively for the purpose of the aforementioned study and in accordance with the relevant legal regulations, in particular the General Data Protection Regulation (GDPR) and the Berlin Data Protection Act (BDSG). The collection and processing of personal data are only lawful with your consent (Article 6 GDPR, § 51 BDSG-new).

By signing the consent form, you agree that the study management and study staff may collect and process your pseudonymized data presented in the participant information for the purpose of the aforementioned study. Except for reactions to adverse events and for the most important steps of the study logistics, your personal data will be processed exclusively in pseudonymized form, meaning that your name will be replaced by a code (e.g., B022). The study leader and study coordinator are responsible for data processing in accordance with the EU General Data Protection Regulation.

Already at the beginning of the study, with the consent form, a code (pseudonym) will be assigned to you as a participant in this study and also to the participating children, under which the storage and evaluation of the data collected in the study will take place. The signed consent forms and all personal data (names, dates of birth, email addresses, telephone

numbers, and postal addresses) remain securely stored in the study center of the Integrative Medicine in Pediatric Oncology at Charité - Universitätsmedizin Berlin. Only the study leader and study coordinator have access to decryption; for all other persons, traceability is not possible. Your data will be treated with care and used only for absolutely necessary study purposes. They will not be published or passed on to third parties. All data are pseudonymously stored on specially secured servers at Charité. They are safe from theft and will not be passed on to third parties not mentioned in this statement.

The study coordinator will pass on the study-related pseudonymized data collected during the study to the responsible study leader Prof. Dr. Georg Seifert and his staff for all matters concerning the practical implementation of the study and the statistical evaluation.

Audio files with personal information will be processed exclusively by the study coordinator by transcribing them without personal information and then analyzing them in a pseudonymized form. The data present at the mentioned points will be pseudonymized and processed and stored for a period of 10 years and then destroyed.

Your Rights as Participants

In

connection with the personal data processed as part of this study, you have the following rights:

You can withdraw your consent to the processing of the data at any time. To do so, please contact the study leader and/or study coordinator (sarah.blakeslee@charite.de). Please note that the legality of the data processing carried out up to that point will not be affected, Article 7 (3) GDPR.

Right to Information: You have the right to information about the personal data concerning you that are collected and processed as part of this study (Article 15 GDPR, §§34 BDSG-new), if this is technically possible.

Right to Rectification: You have the right to have incorrect personal data concerning you corrected (Article 13 GDPR), if this is technically possible.

Right to Erasure: You have the right to have personal data concerning you erased, e.g., if this data is no longer necessary for the purpose for which it was collected (Article 17 GDPR, §§ 35 BDSG-new), if this is technically possible.

Right to Restriction of Processing: Under certain conditions, you have the right to demand the restriction of processing, i.e., the data may only be stored but not processed, if this is technically possible. You must apply for this. Please contact the study leader, study physician, or the data protection officer of the Institute for Social Medicine, Epidemiology and Health Economics, Charité - Universitätsmedizin Berlin for this purpose (Article 18 GDPR).

Right to Object: You have the right to object at any time to specific decisions or measures regarding the processing of personal data concerning you (Article 21 GDPR, § 36 BDSG-new). Such processing will then no longer take place, if this is technically possible.

Deletion of Personal Data:

The personal data will be deleted or destroyed at the latest 10 years after the end of the study.

Publication of Study Results:

The publication of study results will be in anonymized form. Anonymized means that the published results do not allow conclusions to be drawn about your identity or can only be drawn with an disproportionately large amount of time, cost, and effort. We ask for your consent for this step of anonymization, as it constitutes processing within the meaning of Article 4 (2) GDPR. Since after anonymization it is no longer possible to establish a reference to your person, the right to withdraw, information, correction, or deletion is no longer feasible.

If the responsible person for the study-related collection and processing of personal data cannot help you further, you also have the option of contacting the Data Protection Office of Charité - Universitätsmedizin Berlin:

Information on the Processing of Your Personal Data:

The signed consent forms and all personal data (names, dates of birth, email addresses, telephone numbers, and postal addresses) remain securely stored in Integrative Medicine in Pediatric Oncology at Charité - Universitätsmedizin Berlin. Only the study leader and the study coordinator have access to decryption. For all other persons, traceability is not possible. Your data will be treated with care and used only for absolutely necessary study purposes. They will not be published or passed on to third parties.

By signing the consent form, you agree to participate in the study voluntarily. You can withdraw your consent to participate in the study at any time, even if the daycare continues to participate in the study and the daycare Kneipp concept continues to be implemented. The data from the questionnaires and observation protocols will be stored in the study center of the Integrative Medicine working group in the Department of Pediatrics with a focus on oncology/hematology, Charité - Universitätsmedizin Berlin, and evaluated by the study team using statistical and qualitative methods.

The results of the study will be published.

If you have further questions about the study, please feel free to contact the study leader:

Prof. Dr. Georg Seifert, Department of Pediatrics with a focus on Hematology/Oncology/BMT, Charité - Universitätsmedizin Berlin

Charitéplatz 1

10117 Berlin

Telephone: 030/450-DW

Email: georg.seifert@charite.de

For concerns regarding data processing and compliance with data protection requirements, you can also contact the Data Protection Office of Charité - Universitätsmedizin Berlin:

Data Protection Office of Charité - Universitätsmedizin Berlin

Charitéplatz 1

10117 Berlin

Telephone: +49 30 450 580016

Email: datenschutz@charite.de

In the event that you consider data processing to be unlawful, you have the option of lodging a complaint with the supervisory authority responsible for Charité - Universitätsmedizin Berlin:

Berlin Commissioner for Data Protection and Freedom of Information

Friedrichstraße 219

10969 Berlin

We would be delighted to welcome you as a participant in our study!

With warm regards,

Your study team:
Georg Seifert and Sarah Blakeslee

Prompt:
Can you translate this text in English:
Studieninformation Eltern

Version 3 vom 20. September 2022

Integration von Kneippscher Hydrotherapie bei Kindern in Kindertagesstätten - ein Modellprojekt zur Gesundheitsprävention in Berliner Kitas mit wissenschaftlicher Evaluation

Liebe Eltern,

mit dem oben genannten Projekt möchten wir die gesundheitsfördernden Auswirkungen von Kneippschen Wasseranwendungen (Hydrotherapie) in Berliner Kitas (Kindertagesstätte) evaluiert. Ihre Kita möchte an unserem Projekt teilnehmen und damit auch die Zertifizierung als Kneipp Kindergarten durch den Kneipp-Bund e.V. erlangen.

Im Rahmen einer wissenschaftlichen Evaluation des Zertifizierungsprozesses möchten wir untersuchen, inwiefern ein Kneipp Konzept dazu führen kann, dass Berliner Kita Kinder weniger krank werden.

Dafür brauchen wir Ihre Unterstützung als Eltern: Wir bitten Sie, zu Beginn (Herbst 2022) und Ende (Herbst 2023) des Projektjahrs online einige Fragebögen dazu auszufüllen, ob und wie lange Ihr Kind in dieser Zeit krank war.

Zunächst aber stellen wir Ihnen Das Projekt in seinen Grundzügen vor, so dass Sie eine Entscheidungsgrundlage haben:

Hintergründe, Ziele und Nutzen der Studie

In Deutschland hat die Naturheilkunde eine lange Tradition. Der Pfarrer Sebastian Kneipp (1821-1897) gilt als einer der jüngeren Begründer der naturheilkundlichen Medizin und Gesundheitsförderung. Zu den fünf Säulen der Kneippschen Gesundheitslehre gehört die Wasserheilkunde, Kräutermedizin, Bewegung, Ernährung und der Lebensrhythmus. Die fünf Säulen sind in vielen Aspekten bereits mit dem Berliner Kita Bildungsprogramm im Einklang. In vom Kneipp-Bund e.V. zertifizierten Kitas wird dieses Konzept bereits seit über 15 Jahre erfolgreich als Gesundheitsförderung praktiziert.

Kneipp wird heutzutage vor allem mit Wasseranwendungen und der sogenannten „Kneipp-Kur“ in Verbindung gebracht. Die bekanntesten Wassergüsse (z.B. Unterarmguss, Gesichtsguss) und Wickel werden auch bei Kindern angewendet. Es gibt jedoch bisher wenige Studien, die den konkreten gesundheitsfördernden Nutzen des Kneipp Ansatz bei Kita untersuchen.

Ablauf und Dauer der Teilnahme

Wenn Sie an der Studie teilnehmen möchten und die Einverständniserklärung unterzeichnen, erhalten Sie von uns zu Beginn der Studie (ab Herbst 2022) und zum Ende der Studie (Herbst 2023) jeweils für den Zeitraum von 2 Monaten einmal pro Woche per E-Mail einen digitalen Link zu einem kurzen Fragebogen. In diesem Fragebogen werden Sie gebeten anzugeben, ob und wenn ja, wie lange Ihr Kind in der vergangenen Woche krank war. Falls sie die die Fragebogen nicht ausgefüllt haben, bekommen sie am selben Tag eine Erinnerung.

Auf die Einverständniserklärung tragen Sie einmalig Ihre E-Mail-Adresse und Name und Geburtsdatum Ihres Kindes ein. Über die angegebene E-Mail-Adresse senden wir Ihnen über unser Umfragen System ein personalisierter Link zum Fragebogen zu. Die Einverständniserklärung mit Ihren persönlichen Daten wird in einem sicheren Schrank eingeschlossen und ist nur dem Personal der Kita Kneipp Studie zugänglich. Es werden keine weiteren personenbezogenen Daten (Name, Anschrift etc.) erhoben. Um die Daten der verschiedenen Fragebögen zwischen die Kita Fehltage Eingaben und Ihre Angaben zuzuordnen, wird bei der Einwilligung in die Studienteilnahme einen persönlich erstellten Studien Code für Ihren Kind vergeben.

Am Ende der Studie wird an zwei Terminen eine Studie Mitarbeiter*in in die Kita kommen, um zu beobachten, wie das Kneipp Konzept in der Kita umgesetzt wird und ob sich Veränderungen in der Interaktion zwischen die Erzieher*innen und Kinder feststellen lassen. Auch hier werden die protokollierten Beobachtungen komplett ohne persönliche Daten erhoben und auch analysiert.

Freiwilligkeit der Teilnahme

Die Teilnahme an der Datenerhebung im Rahmen der begleitenden Evaluation ist freiwillig und damit unabhängig von der Zertifizierung der Kita durch den Kneipp-Bund e.V.. Es entstehen Ihnen diesbezüglich keine Nachteile, sollten Sie der Datenerhebung nicht zustimmen.

Sie haben jederzeit das Recht, Ihre gegebene Einwilligung zur Studienteilnahme oder zur Weiterverarbeitung Ihrer Daten zu widerrufen und die Studienteilnahme zu beenden.

Risiken der Teilnahme

Ihre Kita möchte sich nach den Leitlinien des Kneipp-Bunds e.V. als Kneipp Kita zertifizieren lassen und wird dieses Konzept in Ihre Kita umsetzten. Kinder können sehr unterschiedlich auf neue Information und Routinen reagieren. Sie werden sanft und behutsam an das Kneipp Konzept nach Kneipp-Bund e.V. Leitlinien herangeführt. Trotzdem kann es sein, dass Ihr Kind nicht an einer Aktivität teilnehmen möchte. In diesem Fall muss Ihr Kind selbstverständlich nicht teilnehmen. Es sind keine weiteren Risiken der Teilnahme zu erwarten. Falls Ihr Kind nicht an die Kneipp Anwendung teilnimmt, werden auch keine weitere Evaluationsdaten erhoben.

Versicherungsschutz

Für diese Studie wurde keine spezielle Versicherung für die Teilnehmenden abgeschlossen. Die an der Studie beteiligten Mitarbeiter*innen der Charité – Universitätsmedizin Berlin sind durch die Betriebshaftpflichtversicherung der Charité gegen Haftpflichtansprüche, welche aus ihrem schuldhaften Verhalten resultieren könnten, versichert.

Datenschutz – Was passiert mit Ihren Daten oder Daten Ihres Kindes?

Die Verarbeitung Ihrer personenbezogenen Daten erfolgt auf der Grundlage der Datenschutzgrundverordnung der EU und des Landesdatenschutzgesetzes Berlin.

Die Erhebung und Verarbeitung der personenbezogenen Daten sowie der Studiendaten erfolgt ausschließlich zum Zwecke der o.g. Studie und folgt gemäß den einschlägigen rechtlichen Bestimmungen, insbesondere der Datenschutzgrundverordnung (DSGVO) und dem Berliner Datenschutzgesetz (BDSG). Die Erhebung und Verarbeitung der personenbezogenen Daten ist nur mit Ihrer Einwilligung rechtmäßig (Artikel 6 DSGVO, § 51 BDSG-neu).

Durch Ihre Unterschrift auf der Einverständniserklärung erklären Sie sich damit einverstanden, dass die Studienleitung und die Studienmitarbeiter*innen Ihre pseudonymisierten Daten zum Zweck der o.g. Studie erheben und verarbeiten dürfen. An allen Stellen außer zur Reaktion bei unerwünschten Ereignissen und für die wichtigsten Schritte der Studienlogistik werden Ihre persönlichen Daten ausschließlich pseudonymisiert verarbeitet, das heißt, dass Ihr Name durch einen Code (z.B. B022) ersetzt wird. Der Studienleiter und Studienkoordinatorin sind die für die Datenverarbeitung Verantwortlichen im Sinne der EU-Datenschutzgrundverordnung.

Schon mit der Einverständniserklärung zu Beginn der Studie wird Ihnen als Teilnehmer*in dieser Studie die von Ihnen eingetragene E-Mail einmalig zum Zweck der Zusendung der Fragebögen erhoben, um eine für Ihnen spezifischer Link in die Fragebogen System zu

erstellen, unter dem die Speicherung und Auswertung der in der Studie erhobenen Daten erfolgt. Die unterschriebenen Einwilligungserklärungen und alle persönlichen Daten (Namen und Geburtsdaten des Kindes, E-Mail-Adresse) verbleiben abgeschlossen in der Integrative Medizin in der pädiatrischen Onkologie an der Charité – Universitätsmedizin Berlin. Nur der Studienleiter und Studie Koordinator*in haben Zugriff auf die Entschlüsselung, für alle anderen Personen ist die Rückverfolgung nicht möglich. Ihre Daten werden sorgsam behandelt und nur an zu unbedingt notwendigen Studienzwecken verwendet. Sie werden keinesfalls veröffentlicht oder an Dritte weitergegeben. Alle Daten werden pseudonymisiert auf speziell gesicherten Servern der Charité gespeichert. Sie sind damit sicher vor Diebstahl geschützt und werden keinesfalls an nicht in dieser Erklärung aufgeführte Dritte weitergegeben.

Die Studienkoordinatorin gibt die während der Studie erhobenen studienbezogenen pseudonymisierten Daten weiter an den verantwortlichen Studienleiter Prof. Dr. Georg Seifert und seine Mitarbeiter*innen für alle Angelegenheiten der praktischen Durchführung der Studie und die statistische Auswertung.

Zusätzlich zu den von Ihnen gemachten Angaben im elektronischen Fragebogen, werden die Kitafehltage in unserem Kita Fragebogen durch die Erzieher*innen dokumentiert. Über den Studiencode Ihres Kindes werden die von Ihnen angegebenen Daten und die der Kita zu Auswertungszwecken verlinkt.

Die bei den genannten Stellen vorhandenen Daten werden pseudonymisiert gespeichert und verarbeitet und für die Zeit von 10 Jahren gespeichert und danach vernichtet.

Ihre Rechte als Teilnehmerinnen

Sie haben im Zusammenhang mit der im Rahmen dieser Studie verarbeiteten personenbezogenen Daten folgende Rechte:

Sie können Ihre erteilte Einwilligung zur Verarbeitung der Daten jederzeit widerrufen. Nehmen Sie hierzu Kontakt mit dem Studienleiter und/oder Studienkoordinatorin (sarah.blakeslee@charite.de) auf. Beachten Sie bitte, dass die Rechtmäßigkeit der bis dahin erfolgten Verarbeitung Ihrer Daten nicht berührt wird, Art. 7 Absatz 3 DSGVO.

Recht auf Auskunft: Sie haben das Recht auf Auskunft über die Sie betreffenden personenbezogenen Daten, die im Rahmen dieser Studie erhoben und verarbeitet werden (Artikel 15 DSGVO, §§34 BDSG-neu), sofern dies technisch möglich ist.

Recht auf Berichtigung: Sie haben das Recht, Sie betreffende unrichtige personenbezogene Daten berichtigen zu lassen (Artikel 13 DSGVO), soweit technisch möglich.

Recht auf Löschung: Sie haben das Recht auf Löschung Sie betreffender personenbezogenen Daten, z. B. wenn diese Daten für den Zweck, für den sie erhoben wurden, nicht mehr notwendig sind (Artikel 17 DSGVO, §§ 35 BDSG-neu), soweit technisch möglich.

Recht auf Einschränkung der Verarbeitung: Unter bestimmten Voraussetzungen haben Sie das Recht, die Einschränkung der Verarbeitung zu verlangen, d. h., die Daten dürfen nur gespeichert, nicht verarbeitet werden, soweit technisch möglich. Dies müssen Sie beantragen. Wenden Sie sich hierzu bitte an den Studienleiter, Studienarzt oder an den

Datenschutzbeauftragten des Instituts für Sozialmedizin, Epidemiologie und Gesundheitsökonomie, Charité – Universitätsmedizin Berlin (Artikel 18 DSGVO).

Widerspruchsrecht: Sie haben das Recht, jederzeit gegen konkrete Entscheidungen oder Maßnahmen zur Verarbeitung der Sie betreffenden personenbezogenen Daten Widerspruch einzulegen (Artikel 21 DSGVO, § 36 BDSG-neu). Eine solche Verarbeitung findet anschließend nicht mehr statt, soweit technisch möglich.

Löschung personenbezogener Daten:

Die personenbezogenen Daten werden spätestens 10 Jahre nach Studienende gelöscht bzw. vernichtet.

Veröffentlichung der Studienergebnisse:

Die Veröffentlichung der Studienergebnisse erfolgt in anonymisierter Form. Anonymisiert bedeutet, dass sich aus den veröffentlichten Ergebnissen keine Rückschlüsse bzw. diese nur mit einem unverhältnismäßig großen Aufwand an Zeit, Kosten und Arbeitskraft auf die Identität Ihrer Person ableiten lassen. Für den Schritt der Anonymisierung erfragen wir Ihre Einwilligung, da es sich um eine Verarbeitung im Sinne von Art. 4 Absatz 2 DSGVO handelt. Da nach einer Anonymisierung ein Bezug zu Ihrer Person nicht mehr herstellbar ist, ist der Anspruch auf Widerruf, Auskunft, Berichtigung oder Löschung nicht mehr durchführbar.

Sollte Ihnen der Verantwortliche für die studienbedingte Erhebung und Verarbeitung personenbezogener Daten nicht weiterhelfen können, haben Sie auch die Möglichkeit mit der Stabsstelle Datenschutz der Charité Kontakt aufzunehmen (siehe Kontakt unten):

Unabhängig davon, dass es Ihnen freisteht, gerichtliche Hilfe in Anspruch zu nehmen, haben Sie das Recht auf Beschwerde bei einer Aufsichtsbehörde, wenn Sie der Ansicht sind, dass die Verarbeitung Ihrer Daten datenschutzrechtlich nicht zulässig ist.

Berliner Beauftragte für Datenschutz und Informationsfreiheit:

Friedrichstr. 219, 10969 Berlin

E-Mail: mailbox@datenschutz-berlin.de

Tel.: 030 13889-0

Beteiligte Personen:

Studienleiter:

Prof. Dr. med. Georg Seifert, Charité - Universitätsmedizin Berlin, Klinik für Pädiatrie m.S. Onkologie/Hämatologie/SZT, AG Integrative Medizin in der pädiatrischen Onkologie

Projektkoordination:

Dr. rer. medic. Sarah Blakeslee, Charité - Universitätsmedizin Berlin, Klinik für Pädiatrie m.S. Onkologie/Hämatologie/SZT, AG Integrative Medizin in der pädiatrischen Onkologie

Wir würden uns sehr freuen, Sie als Teilnehmer/ Teilnehmerin unserer Studie begrüßen zu dürfen!

Mit herzlichen Grüßen,

Ihr Studienteam:
Georg Seifert and Sarah Blakeslee

AI generated answer:
Study Information for Parents

Version 3 dated September 20, 2022

Integration of Kneipp Hydrotherapy for Children in Daycare Centers - A Model Project for Health Prevention in Berlin's Daycares with Scientific Evaluation

Dear Parents,

With the project mentioned above, we aim to evaluate the health-promoting effects of Kneipp water applications (hydrotherapy) in Berlin's daycare centers. Your daycare wishes to participate in our project and thus obtain certification as a Kneipp kindergarten through the Kneipp Association.

As part of a scientific evaluation of the certification process, we aim to investigate to what extent a Kneipp concept can lead to fewer illnesses among children in Berlin daycares.

For this, we need your support as parents: We kindly ask you to fill out some questionnaires online at the beginning (autumn 2022) and end (autumn 2023) of the project year regarding whether and how long your child was ill during this time.

First, however, we will introduce the project in its basic features so that you have a basis for decision-making:

Background, Objectives, and Benefits of the Study

In Germany, naturopathy has a long tradition. The pastor Sebastian Kneipp (1821-1897) is considered one of the younger founders of naturopathic medicine and health promotion. Water therapy, herbal medicine, movement, nutrition, and lifestyle rhythm are among the five pillars of Kneipp's health teachings. The five pillars are already in alignment with many aspects of the Berlin daycare educational program. This concept has been successfully practiced for over 15 years in Kneipp-certified daycares as a means of health promotion.

Kneipp is primarily associated with water applications and the so-called "Kneipp cure" today. The most well-known water treatments (e.g., arm douches, face douches) and wraps are also applied to children. However, there are few studies to date that examine the specific health benefits of the Kneipp approach in daycare settings.

Procedure and Duration of Participation

If you wish to participate in the study and sign the consent form, you will receive from us at the beginning of the study (from autumn 2022) and at the end of the study (autumn 2023) a digital link to a short questionnaire once a week by email for a period of 2 months. In this questionnaire, you will be asked to indicate whether and, if so, how long your child was ill in the past week. If you have not completed the questionnaires, you will receive a reminder on the same day.

You will enter your email address and your child's name and date of birth once on the consent form. We will send you a personalized link to the questionnaire via our survey system to the email address provided. The consent form with your personal data will be securely locked away and accessible only to the personnel of the Kneipp daycare study. No further personal data (name, address, etc.) will be collected. A personally created study code for your child will be assigned when consenting to participate in the study, linking your child's data between the daycare absences entries and your information.

At the end of the study, a study staff member will visit the daycare on two occasions to observe how the Kneipp concept is implemented in the daycare and whether changes in interaction between educators and children can be observed. Again, the recorded observations will be collected and analyzed without any personal data.

Voluntary Participation

Participation in data collection as part of the accompanying evaluation is voluntary and independent of the certification of the daycare by the Kneipp Association. There will be no disadvantages to you if you do not agree to participate in the data collection.

You have the right to withdraw your consent to participate in the study or to further process your data at any time and to end your participation in the study.

Risks of Participation

Your daycare wishes to certify as a Kneipp daycare according to the guidelines of the Kneipp Association and will implement this concept in the daycare. Children may react differently to new information and routines. They will be gently introduced to the Kneipp concept according to the guidelines of the Kneipp Association. Nevertheless, it is possible that your child may not want to participate in an activity. In this case, your child is of course not obliged to participate. No further risks of participation are expected. If your child does not participate in the Kneipp application, no further evaluation data will be collected.

Insurance Coverage

No special insurance has been taken out for participants in this study. The staff involved in the study at Charité - Universitätsmedizin Berlin are insured against liability claims resulting from their negligent behavior by Charité's liability insurance.

Data Protection - What Happens to Your Data or Your Child's Data?

The processing of your personal data is based on the EU General Data Protection Regulation and the Berlin Data Protection Act.

The collection and processing of personal data as well as study data are carried out exclusively for the purpose of the aforementioned study and in accordance with the relevant legal regulations, in particular the General Data Protection Regulation (GDPR) and the Berlin Data Protection Act (BDSG). The collection and processing of personal data are only lawful with your consent (Article 6 GDPR, § 51 BDSG-new).

By signing the consent form, you agree that the study management and study staff may collect and process your pseudonymized data presented in the participant information for the purpose of the aforementioned study. Except for reactions to adverse events and for the most important steps of the study logistics, your personal data will be processed exclusively in pseudonymized form, meaning that your name will be replaced by a code (e.g., B022). The study leader and study coordinator are responsible for data processing in accordance with the EU General Data Protection Regulation.

Already at the beginning of the study, with the consent form, a code (pseudonym) will be assigned to you as a participant in this study and also to the participating children, under which the storage and evaluation of the data collected in the study will take place.

The signed consent forms and all personal data (names and birth dates of the child, email address) will remain securely stored at Integrative Medicine in Pediatric Oncology at Charité - Universitätsmedizin Berlin. Only the study leader and study coordinator have access to decryption. For all other persons, traceability is not possible. Your data will be treated with care and used only for absolutely necessary study purposes. They will not be published or passed on to third parties. All data will be pseudonymized and stored on specially secured servers at Charité. They are safe from theft and will not be passed on to third parties not mentioned in this statement.

The study coordinator will pass on the study-related pseudonymized data collected during the study to the responsible study leader Prof. Dr. Georg Seifert and his staff for all matters concerning the practical implementation of the study and the statistical evaluation.

In addition to the information you provide in the electronic questionnaire, the daycare absences will be documented by the educators on our daycare questionnaire. The data at the mentioned points will be stored and processed in pseudonymized form and will be stored for a period of 10 years and then destroyed.

Your Rights as Participants

You have the following rights in connection with the personal data processed as part of this study:

You can withdraw your consent to the processing of the data at any time. To do so, please contact the study leader and/or study coordinator

. Please note that the legality of the data processing carried out up to that point will not be affected.

Right to Information: You have the right to information about the personal data concerning you that are collected and processed as part of this study, if this is technically possible.

Right to Rectification: You have the right to have incorrect personal data concerning you corrected, if this is technically possible.

Right to Erasure: You have the right to have personal data concerning you erased, e.g., if this data is no longer necessary for the purpose for which it was collected, if this is technically possible.

Right to Restriction of Processing: Under certain conditions, you have the right to demand the restriction of processing, i.e., the data may only be stored but not processed, if this is technically possible. You must apply for this.

Right to Object: You have the right to object at any time to specific decisions or measures regarding the processing of personal data concerning you. Such processing will then no longer take place, if this is technically possible.

Deletion of Personal Data:

The personal data will be deleted or destroyed at the latest 10 years after the end of the study.

Publication of Study Results:

The publication of study results will be in anonymized form. Anonymized means that the published results do not allow conclusions to be drawn about your identity or can only be drawn with a disproportionately large amount of time, cost, and effort.

If the responsible person for the study-related collection and processing of personal data cannot help you further, you also have the option of contacting the Data Protection Office of Charité - Universitätsmedizin Berlin.

Regardless of your right to take legal action, you have the right to lodge a complaint with a supervisory authority if you believe that the processing of your data is not permissible under data protection law.

Berlin Commissioner for Data Protection and Freedom of Information:

Friedrichstr. 219, 10969 Berlin

Email: mailbox@datenschutz-berlin.de

Tel.: 030 13889-0

Involved Persons:

Study Leader:

Prof. Dr. med. Georg Seifert, Charité - Universitätsmedizin Berlin, Department of Pediatrics with a focus on Oncology/Hematology/BMT, Integrative Medicine in Pediatric Oncology

Project Coordination:

Dr. rer. medic. Sarah Blakeslee, Charité - Universitätsmedizin Berlin, Department of Pediatrics with a focus on Oncology/Hematology/BMT, Integrative Medicine in Pediatric Oncology

We would be very pleased to welcome you as a participant in our study!

With warm regards,

Your Study Team:
Georg Seifert and Sarah Blakeslee
