# Supplementary material for: Effect of the health and wellness Kneipp concept on health promotion and reduction of sick days for kindergarten children: a cluster randomized controlled trial protocol
Source: Front Med (Lausanne). 2024 Jul 26;11:1412971. doi: 10.3389/fmed.2024.1412971 (PMC11309992; doi:10.3389/fmed.2024.1412971)
Supplement: Supplementary file 6 [file Data_Sheet_6.PDF]

| Adverse event documentation form |
|---|
| **1. general information** |
| Kita Name: |
| Child's name: |
| Date of birth (DD.MM.YYYY): ☐☐.☐☐.☐☐☐☐ |
| Filled in by:<br>☐ Daycare center management ☐ Other daycare center employees Daycare center educator<br>☐ Parent / legal guardian☐ Other:________________<br><br>Contact details (email and phone): |
| **2. adverse event - symptoms** |
| 2.1 General symptoms |
| **Dizziness**:<br>☐ Light<br>☐ Medium (unsafe to move)<br>☐ Heavy (must lie down) |
| **Tiredness/exhaustion**:<br>☐ Light (requires rest)<br>☐ Medium (exhaustion despite rest)<br>☐ Severe (exhaustion despite rest and requires movement assistance) |

| |
|---|
| **Chills / freezing 20 minutes after Kneipp application and warming up**:<br>☐ Light (trembling; chattering teeth) ☐ Medium (body trembling)<br>☐ Heavy (body trembles constantly) |
| **Pain**:<br>☐ Mild pain<br>☐ Moderate pain (limited activity)<br>☐ Severe pain (restriction of independent activity) |
| **Feeling ill**:<br>☐ Low (discomfort)<br>☐ Moderate symptoms (discomfort or lack of well-being)<br>☐ Severity (discomfort / lack of well-being with restriction of independent activity) |
| **Irritability**:<br>☐ Low (comforting)<br>☐ Moderate symptoms (increased attention indicated)<br>☐ Severe abnormal or excessive reactions (inconsolable) |
| 2.2 Skin |
| **Reddening of the skin**:<br>☐ Mild symptoms<br>☐ Moderate (treatment necessary, everyday life prevented)<br>☐ Severe (hospitalization & restriction of everyday life) |
| **Pallor**:<br>☐ Light (pale/blue >20-30min after treatment)<br>☐ Medium (pale/blue >30-60min after treatment)<br>☐ Severe (pale/blue >60min after treatment) |
| **Rash**:<br>☐ Mild symptoms<br>☐ Moderate (treatment necessary, everyday life prevented)<br>☐ Severe (hospitalization & restriction of everyday life) |

| 2.3 Fall |
|---|
| **Bruise**:<br>☐ Mild symptoms<br>☐ Means (treatment required)<br>☐ Severe (requires hospitalization) |
| **Laceration**:<br>☐ Slight bleeding<br>☐ Medium (Requires medical intervention)<br>☐ Severe (Requires medical intervention and disabled) |
| **Broken bone**:<br>☐ Asymptomatic<br>☐ Symptomatic (entered)<br>☐ Severe (complicated fracture or surgery necessary) |
| 2.4 Breathing |
| **Shortness of breath immediately after a Kneipp treatment**:<br>☐ Shortness of breath with moderate exertion<br>☐ Shortness of breath with minimal exertion<br>☐ Shortness of breath at rest |
| **Rapid breathing which occurs directly after a Kneipp treatment**:<br>☐ Rapid breathing calms down after a short period of sedation<br>☐ Rapid breathing calms down after extended relaxation time<br>☐ Rapid breathing only calms down with medical intervention |
| 2.5 Other symptoms (not mentioned above) |
| ☐ Symptom description:<br>________________________________________<br><br>Detailed description of the severity of the complaint:<br><br>__________________________________________________________________<br>__________________________________________________________________<br>__________________________________________________________________ |

## 3. description of the event:

### 3.1 **Detailed description of what happened**

______________________________________________

______________________________________________

______________________________________________

### 3.2 **Exact time of the event**

Start of the event:

(DD.MM.YYYY)

☐☐.☐☐.☐☐☐☐

Time: _ _ : _ _

End of the event:

(DD.MM.YYYY)

☐☐.☐☐.☐☐☐☐

Time: _ _ : _ _

## 4. documenting person:

Filled in by: ______________________________________________

- ☐ Daycare center management
- ☐ Other daycare center employees
- ☐ Daycare educator
- ☐ Parent / custodian
- ☐ Other:______________________________________________

Contact details

(first name SURNAME):______________________________________________

(email & phone):______________________________________________

______________________________________________
